# Diphasic CeO_2_ Nanocrystal/Bioactive Glass Nanosphere-Based Composite Hydrogel for Diabetic Wound Healing by Reactive Oxygen Species Scavenging and Inflammation Regulation

**DOI:** 10.34133/bmr.0066

**Published:** 2024-09-16

**Authors:** Muyan Qin, Ziyang Zhu, Jingxin Ding, Jinhui Zhao, Lingtian Wang, Dajun Jiang, Deping Wang, Weitao Jia

**Affiliations:** ^1^ School of Materials Science and Engineering, Tongji University, Shanghai 201804, China.; ^2^ Department of Orthopedics, Shanghai Sixth People’s Hospital Affiliated to Shanghai Jiao Tong University School of Medicine, Shanghai Jiao Tong University, Shanghai 200233, China.; ^3^ Department of Orthopedics, Shanghai Tenth People’s Hospital, School of Medicine, Tongji University, Shanghai 200072, China.

## Abstract

**Background:** Antioxidant therapy aimed at reducing excessive local oxidative stress is one of the most important strategies for promoting diabetic wound repair. The reversible transformation of Ce^3+^/Ce^4+^ in ceria (CeO_2_) can reduce excessive local oxidative stress. However, inducing angiogenesis, local anti-inflammatory effects, and other positive effects are challenging. Therefore, ideal dressings for chronic diabetic wound management must concurrently reduce excessive oxidative stress, promote angiogenesis, and have anti-inflammatory effects. **Methods:** In this study, Ce-doped borosilicate bioactive glasses (BGs) were prepared using the sol–gel method, and CeO_2_ nanocrystals (CeO_2_-NCs) were precipitated on the glass surface by heat treatment to obtain BG-*x*Ce composite glass nanospheres. Subsequently, nanospheres were modified by amino group and combined with dopamine and acrylamide to obtain BG-*x*Ce/polydopamine/polyacrylamide (PDA/PAM) composite hydrogel. Then, the morphology and properties of composite hydrogels were detected, and the properties to treat the diabetic wounds were also evaluated. **Results:** The results demonstrated that the BG-10Ce/PDA/PAM composite hydrogel possessed excellent tensile and adhesive properties. In vitro, the migration and angiogenesis of human umbilical vein endothelial cells (HUVECs) and fibroblasts (L929) were enhanced by reducing reactive oxygen species (ROS) levels in the conditioned medium. Animal experiments have shown that CeO_2_-NCs in hydrogels effectively scavenge ROS in diabetic wounds, and Sr dissolved from the glassy phase can modulate macrophage polarization to the M2 phenotype. **Conclusions:** The synergistic effect of both amorphous materials and nanocrystals provides the BG-10Ce/PDA/PAM composite hydrogel with great potential for diabetic wound healing.

## Introduction

Diabetes mellitus is a clinical syndrome characterized by elevated blood glucose levels, affecting the health of millions of people worldwide. Diabetes affects 285 million adults (aged 20 to 79 years) worldwide. This figure is expected to increase to 439 million by 2030 [1,2]. Chronic wounds are common complications in patients with diabetes. These nonhealing wounds are described as being stuck in a persistent inflammatory state characterized by accumulation of proinflammatory macrophages, cytokines, and proteases such that the harsh microenvironment at the wounds disrupts the regenerative function of the skin tissues and makes it difficult to rely on normal physiological functions for healing [3,4]. Diabetes is accompanied by severe chronic skin ulcers and diabetic feet, often leading to amputation [5,6]. Therefore, promoting the healing of diabetic wounds is crucial for the treatment of patients with diabetes.

The skin, which is the largest organ in the body, serves as a defense mechanism against pathogenic invasion, senses the external environment, and regulates the body temperature [7–9]. The complex healing process of skin wounds involves biological tissue reconstruction and repair related to migration and proliferation of fibroblast cells, deposition of extracellular matrix (ECM), and the tissue microenvironment. This process requires 4 stages, such as coagulation, inflammation, migration–proliferation, and remodeling to obtain a more complete repair [10]. However, patients with diabetes have significant healing disorders at all stages of skin healing, which disrupts the tissue healing environment and facilitates the formation of chronic, persistent, nonhealing wounds [5,11].

The hyperglycemic characteristics of patients with diabetes lead to the presence of a large number of advanced glycation end products with strong oxidative properties in the body [12]. This is accompanied by insulin resistance, dyslipidemia, and other factors that easily induce a large amount of reactive oxygen species (ROS) in the mitochondria, including superoxide anions, hydrogen peroxide (H_2_O_2_), and hydroxyl radicals, causing the balance between local pro-oxidation and antioxidation to be tilted toward pro-oxidation. This phenomenon is referred to as oxidative stress [13,14]. Oxidative stress is an important mediator in killing invading bacteria, regulating cellular repair processes, and accelerating tissue repair processes by promoting hemostasis, inflammation, and angiogenesis [15]. However, excessive and uncontrolled oxidative stress causes endothelial tissue damage, reduces angiogenic capacity, induces local inflammatory overexpression resulting in persistent inflammation, generates large amounts of ROS, and accelerates cellular senescence and apoptosis, disrupting the healing process of the skin tissue [5,13]. Therefore, antioxidant therapies aimed at reducing excessive local oxidative stress are important for promoting diabetic wound healing [16,17].

Ceria nanocrystals (CeO_2_-NCs) can function as certain enzymes and are regarded as nanozymes. The reversible transformation of Ce^3+^/Ce^4+^ in the CeO_2_-NCs can reduce local ROS levels and decrease local oxidative stress [18–20]. However, similar techniques lack the ability to promote angiogenesis and ECM deposition, limiting their further application. Recent studies have found that ions dissolved from BG can activate the healing capacity of the wound area (e.g., Cu can up-regulate the expression of angiogenic-related genes and enhance angiogenic capacity [21,22], and Sr can regulate macrophage polarization [23,24]). If CeO_2_-NCs can be precipitated on the surface of Sr/Ce-doped BG to obtain BG-*x*Ce composite glass nanospheres, this material will improve the tissue repair activity of CeO_2_-NCs, reducing oxidative stress, promoting angiogenesis, and regulating macrophage polarization. However, directly covering diabetic wounds with glass nanospheres makes it difficult to control exudation and prevent bacterial invasion [25,26], and is also highly irritating, which cannot provide a mild environment for tissue regeneration [27].

Hydrogel is a wound dressing with a physical structure similar to that of the ECM, which has a soft texture and good moisture retention [28]. Thus, an organic/inorganic composite wound dressing that combines BG-*x*Ce composite glass nanospheres with hydrogels could provide a suitable environment for tissue regeneration in diabetic wounds. Polyacrylamide (PAM) hydrogels are aqueous gels that are stable and nontoxic. They also have mechanical and chemical advantages [29]. However, the adhesive properties of PAM hydrogels are inadequate for use as adhesive layers that adhere to the skin surface [30]. Mussel-inspired hydrogels have shed new light on the development of hydrogels with good cell affinity and tissue adhesiveness. Polydopamine (PDA), inspired by the foot protein of marine blue mussels, has been extensively studied because of its remarkable adhesive properties [29,31,32]. The catechol group of PDA exhibits a high capability for binding diverse substrates (metal, nonmetal, organic, and inorganic substrates), which can be firmly attached to them through reversible noncovalent or irreversible covalent interactions [33,34]. Moreover, PDA-coated materials achieved good cell adhesion, spreading, and high viability in several types of mammalian cells, demonstrating high biocompatibility [35]. The addition of PDA to the PAM hydrogels networks imparts adhesive properties to the resulting PAM/PDA hydrogel owing to the catechol group of PDA [36,37]. PDA/PAM hydrogels, which have high toughness, excellent cell affinity, and tissue adhesion properties, can be used as good carriers of BG-*x*Ce glass nanospheres during diabetic wound repair.

**Fig. 1.**
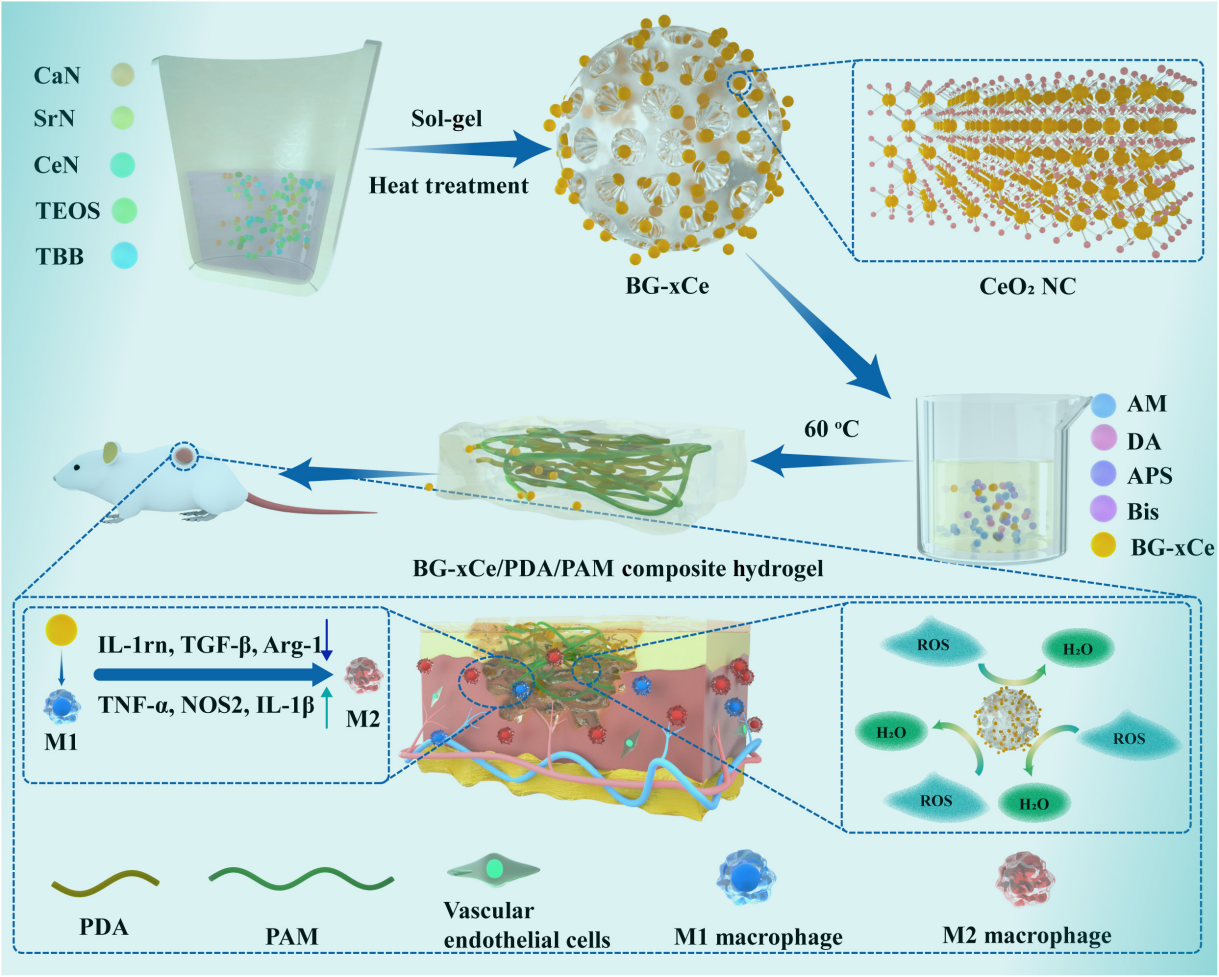
The preparation of BG-*x*Ce/PDA/PAM composite hydrogel and its application on rats with diabetes with full-thickness skin defect.

In this study, we prepared multifunctional hydrogel dressings that effectively improved the microenvironment of diabetic wounds and promoted wound tissue repair by introducing BG-*x*Ce glass nanospheres into PDA/PAM hydrogels. Through heat treatment, CeO_2_-NCs were precipitated on the surface of high-concentration Sr/Ce-doped glass (BG-10Ce), ensuring high adhesion and tensile properties of the composite hydrogels in alkaline environments. In vitro experiments showed that CeO_2_-NCs significantly decomposed H_2_O_2_, reduced ROS levels, and decreased oxidative stress in diabetic wounds. Meanwhile, Sr released from glass nanospheres induced macrophage polarization toward the M2 phenotype and reduced wound inflammation, promoting cell migration and angiogenesis. In vivo experiments also demonstrated that the BG-10Ce/PDA/PAM composite hydrogel accelerated the granulation tissue formation and re-epithelialization, significantly accelerating the healing of chronic diabetic wounds (Fig. 1).

## Methods

### Materials

Tetraethyl orthosilicate (TEOS), tributyl borate (TBB), calcium nitrate tetrahydrate (CaN), cerium(III) nitrate hexahydrate (CeN), strontium nitrate (SrN), cetyltrimethylammonium bromide (CTAB), silane coupling agent KH-550, dopamine hydrochloride (DA), acrylamide (AM), *N*,*N*′-methylenebisacrylamide (BIS), ammonium persulfate (APS), *N*,*N*,*N*′,*N*′-tetramethylethylenediamine (TEMDA), ammonia water, hydrogen peroxide aqueous solution (H_2_O_2_), titanium(IV) sulfate, sulfuric acid, ethanol absolute, and streptozotocin (STZ) were all analytically pure reagents and purchased from Sinopharm Chemical Reagent Co. Ltd., Shanghai, China. Dulbecco’s modified Eagle’s medium (DMEM), fetal bovine serum (FBS), and penicillin/streptomycin (P/S) were purchased from Beyotime Biotechnology. Endothelial cell culture medium (ECM) with endothelial cell growth supplement (ECGS) was purchased from ScienCell Research Laboratories.

### Preparation of BG-*x*Ce composite glass nanospheres

The chemical composition of the BG-*x*Ce composite glass nanospheres is shown in Table 1, where SiO_2_ is introduced by TEOS, B_2_O_3_ by TBB, CaO by CaN, and CeO_2_ by CeN. The composite glass nanospheres were prepared by sol–gel method [27,38], and the specific preparation steps were taken as an example for 70SiO_2_-10B_2_O_3_-10CaO-5SrO-5CeO_2_: 1.458 g of CTAB and 6.7 ml of ammonia solution (25 wt%) were dissolved in 400 ml of deionized water at 40 °C. After the solution became clear and uniform, a mixture of 5.6 ml of TEOS and 1.9 ml of TBB was added dropwise and magnetically stirred vigorously. Then, 0.84 g of CaN aqueous solution (2 ml) and 0.772 g of CeN aqueous solution (2 ml) were added dropwise every 30 min, and the reaction was continued for 4 h. The particles were centrifuged and washed twice with ethanol and deionized water. The obtained samples were freeze-dried and subsequently sintered at 600 °C for 180 min (heating rate of 2 °C/min) to obtain the BG-*x*Ce composite glass nanospheres.

### Surface modification of BG-*x*Ce composite glass nanospheres

Amino groups were grafted onto the surface of the nanospheres to improve the dispersion of the BG-*x*Ce composite glass nanospheres and to make them more uniformly dispersed in the hydrogel. The process was as follows: 2 g of composite glass nanospheres was dispersed in 100 ml of ethanol, and 4 ml of deionized water and 4 ml of silane coupling agent KH-550 were added into solution, which was afterward stirred at 60 °C for 12 h. Finally, nanospheres were centrifuged, washed twice with deionized water and once with ethanol, and freeze-dried to obtain the modified nanospheres.

### Preparation of BG-*x*Ce/PDA/PAM composite hydrogel

The compositions of the BG-*x*Ce/PDA/PAM composite hydrogels are listed in Table 2. The specific preparation steps were as follows: 50 mg of amino-modified BG-*x*Ce composite glass nanospheres was dispersed in 30 ml of deionized water and sonicated for 15 min to obtain a stable suspension. Then, 0.0168 g of DA and 5.6 g of AM were added to the suspension, and 0.02 g of BIS, 1.29 g of APS, and 20 μl of TEMDA were added after complete dissolution of DA and AM. The composite solution was stirred for 10 min and subsequently transferred to a mold, which was then heated in a sealed oven at 60 °C for 2 h to obtain the designed shape of the BG-*x*Ce/PDA/PAM composite hydrogel (*x* = 0, 5, and 10 mol%).

### Characterization of BG-*x*Ce composite glass nanospheres

The structure of the BG-*x*Ce composite glass nanospheres was characterized using x-ray powder diffraction (XRD, D/max2550VB3+/PC, Rigaku International Co., Japan) with a scanning speed of 5°/min in the 2θ ranges from 10° to 80°. The morphology of the samples was observed using scanning electron microscopy (SEM; Hitachi S-4700, Japan) and transmission electron microscopy (TEM; JEM-2100F, JEOL, Japan). Moreover, the elemental composition was determined using energy-dispersive spectroscopy (EDS). Nitrogen adsorption–desorption isotherms were measured using a specific surface area (SSA) and pore size analyzer (NOVA 2200e, Quantachrome, USA). BG-*x*Ce composite glass nanospheres were dried in an oven at 60 °C for 24 h and then degassed at 100 °C to test the SSA and pore size distributions.

The BG-*x*Ce composite glass nanospheres were immersed in phosphate-buffered saline (PBS) and tested for ion release using inductively coupled plasma optical emission spectrometry (ICP-OES; ICP-8300, PerkinElmer, USA) to evaluate their degradation properties: Composite glass nanospheres (50 mg) (BG-0Ce, BG-5Ce, and BG-10Ce) were added to 10 ml of PBS; 1 ml of the immersion solution was removed at 1, 3, 5, and 7 d for ICP-OES analysis to determine the concentrations of various ions in the soaking solution; and 1 ml of fresh PBS was added back to the immersion solution.

### Antioxidant properties of BG-*x*Ce composite glass nanospheres

X-ray photoelectron spectroscopy (XPS; ESCALAB 250Xi, Thermo Fisher Scientific, USA) experiments was performed using an ESCALAB 250Xi photo electron spectrometer equipped with a standard Al anode. The fine Ce spectra were deconvoluted using the Casa XPS software to examine the valence distribution of Ce.

The method reported in [39] was used to evaluate the H_2_O_2_ scavenging capacity of the BG-*x*Ce composite glass nanospheres. Additionally, 50 mg of composite glass nanospheres (BG-0Ce, BG-5Ce, and BG-10Ce) was added to 10 ml and 0.075 M H_2_O_2_ solution. After a specific reaction time, 100 μl of H_2_O_2_ solution and 200 μl of Ti(SO_4_)_2_ solution were taken into 96-well plates and the absorbance of the solution at 405 nm was measured to evaluate the concentration of H_2_O_2_.

Superoxide anion (•O^2−^) radical scavenging capacity was evaluated using a superoxide dismutase mimic (SOD) assay kit (BC0175, Solarbio, China) [14,40], following the provided instructions. BG-*x*Ce nanospheres were immersed into working solution for 30 min at 37 °C. The absorbance at 560 nm was detected using a microplate reader, and inhibition percentage of SOD was calculated.

### Characterization of BG-*x*Ce/PDA/PAM composite hydrogel

The BG-*x*Ce/PDA/PAM composite hydrogels were prepared as cylinders, and the freeze-dried samples were used for SEM to observe the surface morphology and perform elemental analysis using EDS. The chemical groups of the hydrogels were examined using an attenuated total reflection Fourier transform infrared spectrometer (ATR-FTIR; Nicolet iS20, Thermo Fisher Scientific, USA).

The hydrogels were prepared as rectangular bodies for tensile property testing (*n* = 5) at a stretching rate of 500 mm/min and a load of 5 kN at room temperature. Young’s modulus was obtained by calculating the slope of curve in the scope of strain from 0 to 10%. The maximum tensile ratio *λ*_*c*_ reflects the tensile properties of the material and can be calculated as follows:

$$\lambda_{c}=\frac{l_{m}}{l_{0}}$$ (1)

where *l_m_* is the maximum stretch length and *l*_0_ is the original hydrogel lengths.

The swelling ratio of hydrogels was tested based on the method in [41]. The hydrogels were freeze-dried, and the initial weight (*m*_*0*_) was recorded. The dried samples were then soaked in deionized water for 3 d until equilibrium was reached. The final weight of the hydrogels (*m*_*1*_) was determined after removing excess water from the surface using an absorbent paper. The swelling ratios of hydrogels are expressed as follows:

$$\text{Swelling ratio}\;\;\left(\%\right)=\frac{m_{1}-m_{0}}{m_{0}}*100\%$$ (2)

Fresh porcine skin was used as the substrate, and the adhesive properties of the hydrogel on the substrate were tested by macroscopic adhesion tests using universal testing machine (UTM2502, Suns, China). First, the porcine skin was cut into squares (20 × 20 mm), which were then bonded to the skin and pressed at room temperature for a few minutes. Finally, the adhesive strength of the hydrogels (*n* = 5) was tested at a constant rate of 5 mm/min.

### In vitro cellular experiments with BG-*x*Ce/PDA/PAM composite hydrogels

#### Cell culture

All the cells were obtained from the Type Culture Collection of the Chinese Academy of Sciences (Shanghai, China). Mouse fibroblasts (L929) and mouse mononuclear macrophage leukemia cells (RAW 264.7) were cultured in complete DMEM (10% FBS + 1% P/S). Human umbilical vein endothelial cells (HUVECs) were cultured in ECM (ScienCell, USA) (5% FBS + 1% P/S + 1% ECGS). All cells were incubated in a humidified 5% CO_2_ incubator at 37 °C.

#### Cytocompatibility

Composite hydrogel extracts were prepared according to the ISO 10993-12-2021 standard [42]. Based on the swelling test results, an appropriate amount of PBS was added to allow the hydrogel to reach its swelling limit. The base medium was added to hydrogel soaking solution at the ratio of 0.1 g/ml, which was then placed on a slow shaker at 37 °C for 72 h. The extracts were collected, filtered using 0.22-μm filter, and stored at 4 °C for backup. A complete medium was prepared before using it according to a ratio of 10% FBS and 1% P/S.

Each group of hydrogel extracts was diluted proportionally with complete DMEM. L929s were cultured in 96-well plates at an initial seeding density of 5 × 10^3^ cells/well (*n* ≥ 3). When cells were fully adherent, the culture medium was replaced with diluted hydrogel extracts, and complete DMEM was used as the control group. After 24 h of culture, CCK-8 reagent was added to each well, and the absorbances was measured at 450 nm using a microplate reader (Bio-Rad 680, USA). The relative growth rate (RGR) was calculated as follows:

$$\mathrm{RGR}=\frac{A_{t}-A_{0}}{A_{c}-A_{0}}\times 100\%$$ (3)

where *A_t_*, *A_c_*, and *A*_0_ are the absorbance of the experimental, control, and blank groups, respectively.

#### Intracellular ROS analysis

RAW 264.7 cells were cultured in 24-well plates at an initial seeding density of 5 × 10^4^ cells/well (*n* ≥ 3). When cells were fully adhered, lipopolysaccharide (LPS) (100 ng/ml) was added to produce endogenous ROS after 8 h. Then, hydrogels from each group were added and cocultured with the cells. The group without LPS induction was set as the negative control group, and the LPS induction group was set as the positive control group. After 24 h of incubation, intracellular ROS levels were detected using the fluorescent probe 2′,7′-dichlorodihydrofluorescein diacetate (DCFH-DA) according to the method of the ROS detection kit (S0033S, Beyotime, China), which was observed using a fluorescence microscope (DMi8, Leica, Germany) and photographed. The ImageJ software was used to analyze the average fluorescence intensity of each group.

### Regulation of macrophage polarization by BG-*x*Ce/PDA/PAM composite hydrogels

#### Flow cytometry

RAW 264.7 cells were cultured in 24-well plates at an initial seeding density of 5 × 10^4^ cells/well (*n* ≥ 3). When cells were fully adhered, LPS (100 ng/ml) was added for 8 h, and the culture medium was replaced with the diluted hydrogel extracts of each group. The group without LPS induction was used as the negative control, and the LPS induction group was used as the positive control. After 24 h of incubation, cells were collected and labeled with flow cytometry antibodies for the M1 macrophage surface marker CD86 (allophycocyanin anti-mouse CD86 antibody, BioLegend, CA, USA) and the M2 macrophage surface marker CD206 (phycoerythrin anti-mouse CD86 antibody, BioLegend). The Guava easyCyte Flow Cytometer (Millipore, USA) and CytExpert software were used to perform flow cytometry assays and data analysis, respectively.

#### Immunofluorescence staining

Cells were cultured (*n* ≥ 3), grouped as previously described, and fixed in 4% paraformaldehyde (PFA) at room temperature for 30 min. Immunostaining closure solution (P0102, Beyotime) was used for blocking and permeabilization at room temperature for 1 h. Primary antibodies against inducible nitric oxide synthase (iNOS; Abcam, 1:500) and arginase-1 (Arg-1, Abcam, 1:200) were added into wells and incubated overnight at 4 °C. Secondary Goat Anti Rabbit Alexa Fluor 488 (Abcam, 1:200) and Alexa Fluor 594 (Abcam, 1:200) antibodies were added into wells for 1 h to bind iNOS and Arg-1 primary antibodies, respectively. The cytoskeleton was labeled with phalloidin (Yeasen, Shanghai, China), and the nucleus was labeled with 4′,6-diamidino-2-phenylindole (DAPI) (Beyotime). A fluorescence microscope (DMi8, Leica) was used to observe and photograph the cells, and the average fluorescence intensity of each group of iNOS and Arg-1 cells was analyzed using the ImageJ software.

#### Quantitative reverse transcription polymerase chain reaction

RAW 264.7 cells were cultured in 6-well plates at an initial seeding density of 5 × 10^5^ cells/well (*n* ≥ 3), and the rest of the operation was the same as above. After 24 h of incubation, RNA was extracted from the cells using an RNA extraction kit (B0004D, EZBioscience, China) and reverse transcribed to cDNA using a reverse transcription kit (A0010CGQ, EZBioscience). Then, quantitative polymerase chain reaction (qPCR) kit (A0012-R2, EZBioscience) was used to evaluate the relative expression level of proinflammatory genes [interleukin-1β (IL-1β), tumor necrosis factor–α (TNF-α), nitric oxide synthase 2 (NOS2)] and anti-inflammatory genes [IL-1rn, transforming growth factor-β (TGF-β), Arg-1] in each group of cells. Table 3 lists the primers used in these experiments.

### Effect of hydrogels on cell migration and tube formation

#### Preparation of macrophage conditioned medium

After coculturing with LPS-induced macrophages for 24 h, the supernatant was collected from the BG-*x*Ce/PDA/PAM composite hydrogels. The medium was filtered using a 0.22-μm filter and mixed with fresh medium at a ratio of 1:2 (v/v) to obtain the macrophage conditioned medium (MCM) of each group, which was stored at 4 °C.

#### Scratch assay

L929s were inoculated into 6-well plates at an initial seeding density of 5 × 10^4^ cells/well (*n* ≥ 3). A 200-μl pipette tip was used to make a scratch in the center of the well when 80% fusion was achieved, followed by washing with PBS buffer. MCM from each group with reduced serum concentrations was added to each well. After incubation for 24 h, cells were fixed with 4% PFA at room temperature for 30 min, stained with crystal violet at room temperature for 30 min, and then observed under a microscope (Ts2R, Nikon, Japan). The scratch area was quantified using the ImageJ software. Scratch closure rate (SCR) was calculated using the following equation:

$$\mathrm{SCR}\;\;\left(\%\right)\;=\frac{S_{0}-S_{1}}{S_{0}}\times 100\%$$ (4)

where *S*_*0*_ and *S*_1_ represent the scratch area of 0 and 24 h, respectively.

#### Cell migration assay

HUVECs were cultured in the upper chamber of transwell (8 μm, Corning, USA) at an initial seeding density of 5 × 10^4^ cells/well (*n* ≥ 3), and each group of MCM was added into bottom chambers. After culturing for 24 h, the cells were fixed with 4% PFA at room temperature for 30 min and stained with crystal violet at room temperature for 30 min. The cells that migrated to the lower side were retained, as observed under an optical microscope (Eclipse Ts2R, Nikon, Japan), and counted.

#### Angiogenesis assay

Angiogenic slides (ibidi) with the Matrigel (Corning, USA) were commonly used to study the angiogenic capacity of endothelial cells. The Matrigel (10 μl) was added to the bottom chamber of the ibidi angiogenesis slide and cured at 37 °C for 15 min. Then, HUVECs were inoculated onto the Matrigel, cultured with each group of MCM, and cultured at 37 °C for 6 h (*n* ≥ 3). Cells were fluorescently stained with calcein. A fluorescence microscope (DMi8, Leica) was used to observe the HUVECs on the surface of the Matrigel in a vascularized meshwork and photographed.

### In vivo animal study of composite hydrogel

All animal experiments were performed based on the regulations of animal experimental management methods of Shanghai Sixth People’s Hospital and approved by the Animal Ethics Committee of Shanghai Sixth People’s Hospital with permit number 2022-0595. Sprague–Dawley (SD) male rats were housed in a specific pathogen-free (SPF) environment at 3 rats per cage with room temperature maintained at ~22 to ~27 °C, 12/12 h diurnal rule. All rats were fed and watered freely.

#### Type I diabetes model in SD rats

First, the fasting plasma glucose levels of SD rats were measured and recorded, STZ solution was intraperitoneally injected, and the blood glucose levels of all SD rats were tested at 3, 7, 10, and 14 d after surgery. If the blood glucose value was exceeded 16.7 mM (300 mg/dl) for 3 consecutive times, the rats were considered to have successfully established type I diabetes mellitus. All SD rats with successfully established type I diabetes were randomly divided into 4 groups (*n* > 12), including the control group, BG-0Ce group, BG-5Ce group, and BG-10Ce group, respectively.

#### Wound healing of diabetes SD rats with full-thickness skin defects

First, the SD rats were anesthetized, and their dorsal hair was shaved. Subsequently, the rats were fixed in the prone position, a circular mark with a diameter of 2 cm was made at the center of the back, and the whole skin of the rats was excised along the mark after sterilization. The wound was rinsed with saline, and gauze pressure was applied to stop bleeding until no apparent bleeding was observed. Using hydrogels without the BG-*x*Ce composite glass nanospheres as controls, each group of hydrogels was covered and confirmed to adhere to the wounds. After 3, 7, 14, and 21 d of treatment, the wounds were photographed to record their size. ImageJ was used to analyze the images and calculate the wound healing rate of each group of rats. The wound healing rate was calculated using Eq. 5:

$$\text{Wound healing rate }\left(\%\right)\;=\frac{A_{0}-A_{t}}{A_{t}}\times 100\%$$ (5)

where *A*_0_ represents the wound size at 0 day and *A_t_* represents the wound size at *t* day (*t* = 3, 7, 14, and 21).

#### Histological analysis

After curing for 7 d, the SD rats were sacrificed to harvest the original surface and the surrounding 5 mm of full skin tissue. Subsequently, the skin tissue was embedded in optimal cutting temperature (OCT) and snap frozen in liquid nitrogen to promote coagulation. Frozen tissue sections were obtained and used for immunofluorescence staining.

After curing for 7, 14, and 21 d, the SD rats were sacrificed to harvest the original surface and the surrounding 5 mm of full skin tissue. Subsequently, the skin tissue was fixed with 4% PFA overnight and embedded in paraffin blocks to obtain paraffin tissue sections for subsequent histological and immunofluorescence staining.

Hematoxylin and eosin (H&E) and Masson’s trichrome staining were used to analyze wound repair and collagen deposition and arrangement. Sirius red staining was used to study the arrangement and composition of the different collagen type in the regenerated skin tissues. For in vivo superoxide level analysis, frozen tissue sections were stained with dihydroethidium (5 μM) and observed using the fluorescence microscope (DMi8, Leica). Moreover, the immunofluorescence staining of iNOS and CD206 was performed to evaluate the modulating effect of composite hydrogel on macrophage polarization. The immunofluorescence staining of collagen I and collagen III was performed to evaluate the wound closure. Meanwhile, the immunofluorescence staining of CD31 and α-SMA (smooth muscle actin) was performed to evaluate the angiogenesis of wound, and the number of neovascularization was statistically analyzed.

### Statistical analysis

All statistical analyses were performed using the GraphPad Prism 8 software (GraphPad Software Inc., La Jolla, CA, USA). Results were presented as the mean ± standard deviation, with differences between groups analyzed using one-way analysis of variance (ANOVA), followed by Tukey’s multiple comparisons test. Statistical significance was set at *P* < 0.05.

## Results

### Characterization of BG-*x*Ce composite glass nanospheres

**Fig. 2.**
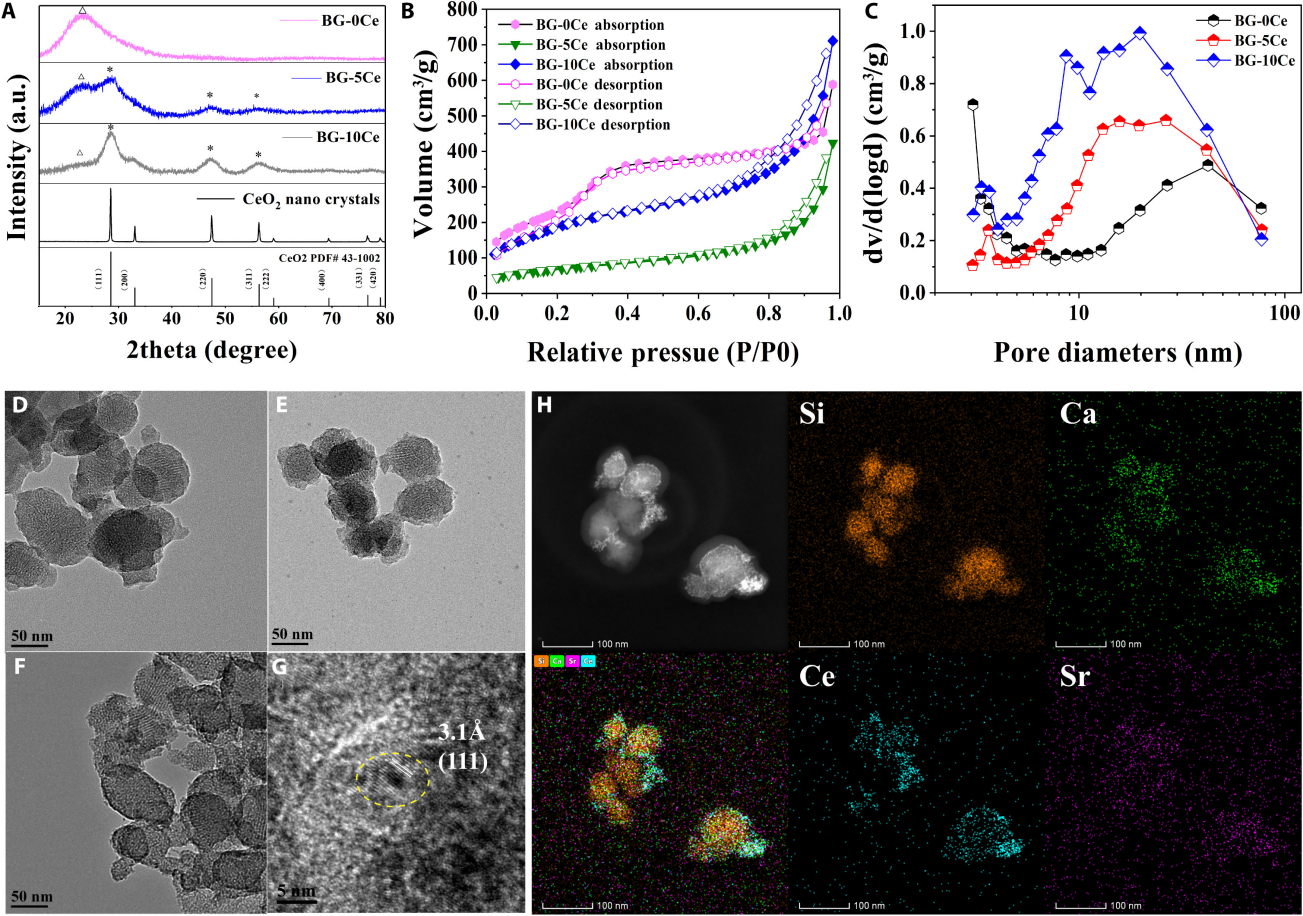
Characterization of BG-*x*Ce (*x* = 0, 5 ,10 mol%) composite glass nanospheres. (A) XRD patterns of the prepared 3 types of nanospheres. (B) Nitrogen adsorption and desorption isotherms and (C) pore size distribution of the prepared 3 types of nanospheres. (D to F) TEM images of the prepared nanospheres. (G) HRTEM images of the BG-10Ce nanospheres. (H) High-angle annular dark-field scanning TEM image and elemental mapping images (Si, Ca, Ce, and Sr) of BG-10Ce nanospheres (scale bar, 100 nm).

The XRD patterns of BG-*x*Ce (*x* = 0, 5, 10 mol%) composite glass nanospheres are shown in Fig. 2A, while patterns of BG-0Ce displayed broad peaks from 20° to 30°, indicating the amorphous nature of glass. In the BG-5Ce composite glass nanospheres, sharp diffraction peaks progressively appeared, corresponding to the (111), (220), and (311) crystal planes of CeO_2_. The intensity of the diffraction peak of the (111) crystal planes was higher than that of the broad peak. In the BG-10Ce composite glass nanospheres, the diffraction peak position remained unchanged, and the intensity of the diffraction peak further increased, resulting in the narrowing of the half-height and appearance of the peak position of the (200) crystal planes, which corresponded to the peak position of CeO_2_ (PDF#43-1002). Figure S1 shows the SEM micromorphology of the BG-*x*Ce composite glass nanospheres. Glass nanospheres were observed to be relatively homogeneous in size and retained a decent spherical shape as the concentration of Ce increased. The EDS analysis of the BG-10Ce group revealed that the Ce content on the surface of the BG-10Ce nanospheres was elevated. Figure 2D to F illustrates the TEM images of the BG-*x*Ce composite glass nanospheres, in which the BG-0Ce glass nanospheres had a regular pore size structure, and the surfaces of the nanospheres were smooth and contrasted uniformly across the field of view. The BG-5Ce and BG-10Ce groups also had regular pore size structures; however, there was a significant increase in contrast on the surface of the nanospheres, indicating the deposition of substances with substantial relative atomic weights on the surface. Moreover, the high-resolution TEM (HRTEM) images of the BG-10Ce group featured distinct lattice fringe spacings of 3.1 Å, which were assigned to the (111) lattice planes of CeO_2_. In conjunction with the XRD results, it was demonstrated that the material deposited on the surface of the BG-10Ce glass nanospheres was CeO_2_ (Fig. 2G). Further, the elemental composition analysis using energy-dispersive X-ray spectroscopy (EDS) showed that the Si, Ca, Ce, and Sr elements existed in the composite glass nanospheres (Fig. 2H). Because B has a low relative atomic mass, results of EDS have a large error. Therefore, the presence of B was proved by the results of ICP-OES (Fig. S2B).

Figure S3 shows the particle distribution of the BG-*x*Ce composite glass nanospheres fitted with the Gaussian function, of which the diameters were 68.95 ± 0.86 nm, 66.42 ± 0.33 nm, and 63.99 ± 0.95 nm for BG-0Ce, BG-5Ce, and BG-10Ce, respectively.

The nitrogen adsorption and desorption profiles, as well as the pore size distribution, indicated that all the samples possessed a mesoporous structure (Fig. 2B and C). The average pore sizes of the composite glass nanospheres were 3.87, 10.83, and 6.48 nm, respectively. The SSAs were 939, 241, and 678 m^2^/g, and the total pore volumes were 0.91, 0.65, and 1.10 cm^3^/g. After the introduction of Ce, the micropores were progressively filled with a sudden decrease in the proportion and a gradual increase in the number of mesopores.

The BG-*x*Ce composite glass nanospheres were immersed in a PBS solution, and their degradation characteristics were analyzed using ICP-OES. The nanospheres progressively released B, Si, Sr, and Ce ions when immersed in the PBS solution, as shown in Fig. S2A to D. The release rates of B, Sr, and Ce in the BG-10Ce group were higher than those in the other groups, suggesting that the addition of Ce in the glass composition decreased the stability of the glass network structure.

### Antioxidant properties of BG-*x*Ce composite glass nanospheres

The distribution of Ce^3+^/Ce^4+^ in glass nanospheres cannot be overstated, as it plays a crucial role in the scavenging of oxidative stress; therefore, it is necessary to conduct detailed peak splitting of the XPS fine spectra of Ce. The detection signal was random since the BG-0Ce group lacked Ce (Fig. S4A). The fine spectra of BG-5Ce and BG-10Ce were a sight to behold, showcasing the classical energy spectral peak positions of Ce. The relative proportions of Ce^3+^ in BG-5Ce were 26.79%, with Ce^4+^ making up the remaining 73.21% (the ratio of Ce^3+^/Ce^4+^ = 36.59%), whereas they were 29.26% and 71.74% in BG-10Ce (the ratio of Ce^3+^/Ce^4+^ = 40.79%).

To verify the antioxidant properties, the H_2_O_2_ scavenging capacity and •O^2−^ scavenging capacity of BG-*x*Ce nanospheres were evaluated. Figure S5A illustrates that all the samples had H_2_O_2_ scavenging capacity during 84 h, where BG-10Ce nanospheres had the best H_2_O_2_ scavenging capacity compared to the other groups. Meanwhile, results in Fig. S5B proved that nanospheres had SOD-mimic activity of scavenging •O^2−^ radical, and BG-10Ce nanospheres had the highest •O^2−^ inhibition rate at 57%. These results collectively demonstrated that BG-10Ce nanospheres have excellent scavenging ability of diverse ROS in all the groups.

### Characterization of BG-*x*Ce/PDA/PAM composite hydrogels

**Fig. 3.**
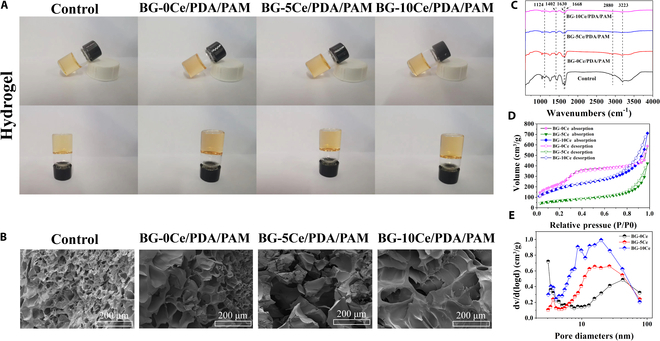
(A) Photograph of BG-*x*Ce/PDA/PAM composite hydrogels. (B) Pore morphology of BG-*x*Ce/PDA/PAM composite hydrogels. (C) ATR-FTIR of BG-*x*Ce/PDA/PAM composite hydrogels. (D) Tensile stress–strain curve and (E) adhesive curve of BG-*x*Ce/PDA/PAM composite hydrogels.

The appearance of the BG-*x*Ce/PDA/PAM composite hydrogel was shown in Fig. 3A. After 2 h at 60 °C, a homogeneous and transparent hydrogel was produced by a cross-linking reaction. When inclined and inverted, the cross-linked hydrogel did not exhibit an apparent flow phenomenon. Figure 3B shows the SEM image of the composite hydrogels after freeze-drying, along with elemental mapping of the BG-10Ce/PDA/PAM group. In the control group, hydrogel pores were dense, small in diameter, and uniformly distributed. In the BG-*x*Ce/PDA/PAM group, the pore size of the hydrogels progressively increased, with no agglomeration of BG-*x*Ce nanospheres observed in the hydrogels. The EDS energy spectrum analysis (Fig. S6) in the BG-10Ce/PDA/PAM group revealed that Si, Ca, Ce, and Sr in the glass nanospheres were uniformly distributed, indicating that the glass nanospheres were uniformly dispersed in the hydrogels without any stacking. The ability of hydrogels to absorb large amounts of fluid for decomposition facilitates wound healing; therefore, the ability of the wound dressing to absorb exuded tissue fluid at the site of the wound is crucial. The swelling rates of the freeze-dried samples were 216 ± 15.1% (control group), 223 ± 13.5% (BG-0Ce/PDA/PAM group), 237 ± 10.5% (BG-5Ce/PDA/PAM group), and 302 ± 10.5% (BG-10Ce/PDA/PAM group). This was primarily because after the addition of glass nanospheres, the pores of hydrogels gradually increased and their water absorption increased, thereby enhancing their swelling performance.

Figure 3C demonstrates the ATR-FTIR of hydrogels. The peaks at 1,630 cm^−1^ were assigned to the primary amines of PDA, indicating that DA was successfully polymerized in hydrogels. The peaks at 1,668 and 1,402 cm^−1^ correspond to the C═O stretching of PAM and C–N stretching of the primary amide cross-linked by PDA and PAM, respectively. The peaks at 1,124 cm^−1^ were assigned to the -NH_2_ rocking of PAM, and the results revealed the formation of PDA and PAM interconnected by a primary amide. Figure 3D and E shows the tensile and adhesive properties of the hydrogels. The tensile properties of the composite hydrogels were enhanced by the incorporation of the BG-*x*Ce nanospheres into the PDA/PAM hydrogel matrix, as shown in Fig. 3D. The breaking strength increased from 4 kPa (control group) to 18 kPa (BG-10Ce/PDA/PAM group), and the tensile deformation increased from 2.2 to 5.5 times. Young’s modulus was calculated and shown in Fig. S7A. This remarkable increase in the tensile properties could be explained by the fact that the -NH_2_ group at the modified nanospheres surface provided more cross-linking sites and enhanced the overall structural integrity of the hydrogels. However, the maximum adhesion forces of the hydrogels differently, as shown in Fig. 3E and Fig. S7B. The maximum adhesion force was 63.77 kPa (control group), 46.5 kPa (BG-0Ce/PDA/PAM group), 56.3 kPa (BG-5Ce/PDA/PAM group), and 60.84 kPa (BG-10Ce/PDA/PAM group), respectively. This could be explained by the oxidation of the hydroxyl group at the benzene ring by the alkaline environment caused by the degradation of the glass nanospheres, leading to a decrease in the maximum adhesion force.

### In vitro cell experiments and ROS scavenging ability

**Fig. 4.**
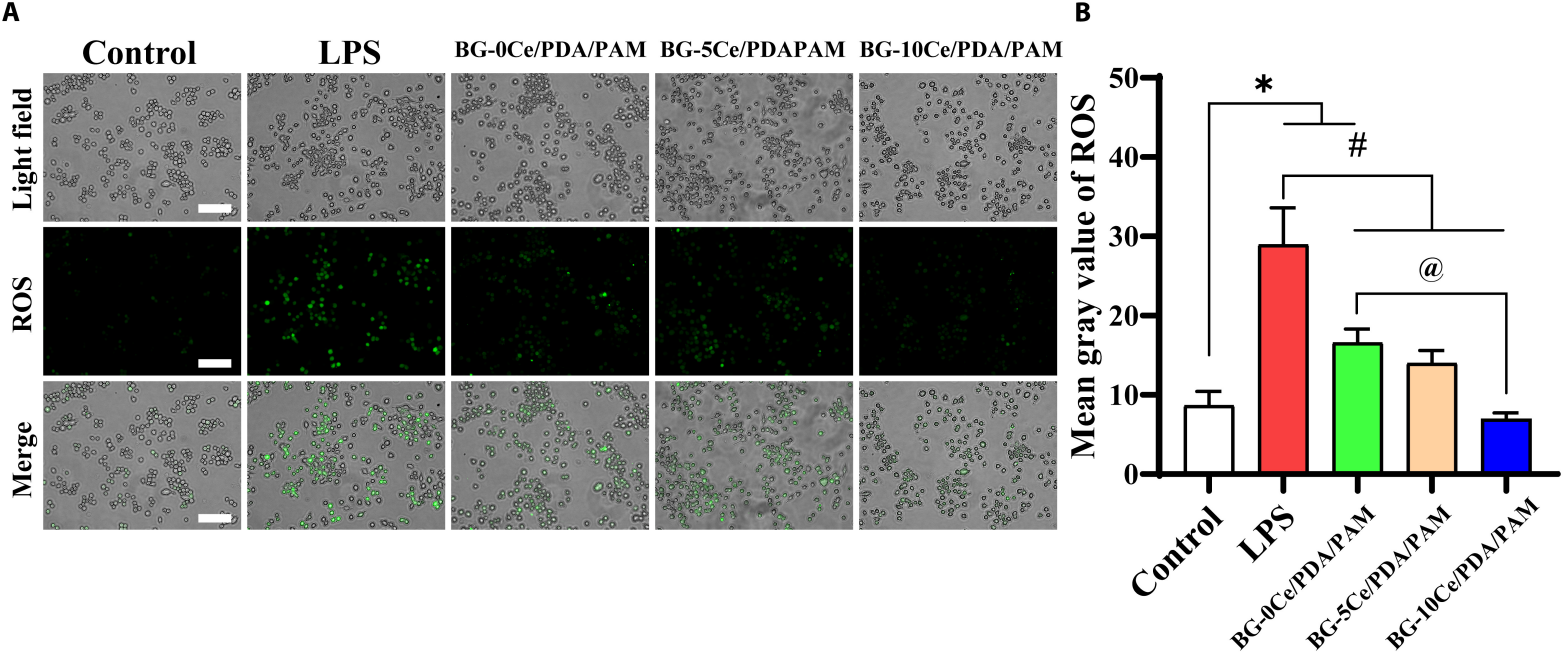
(A) Fluorescent staining for intracellular ROS scavenging (scale bar, 100 μm) and (B) quantitative statistics of fluorescence intensity (*P, ^#^*P*, ^@^*P* < 0.05, *n* = 5).

The extracts of BG-*x*Ce/PDA/PAM composite hydrogels were diluted to 1:2, 1:4, and 1:8 concentrations and cocultured with L929s. The proliferation of L929s in each group was detected using CCK-8. As shown in Fig. S8, with a mean value of 1 for the control group, the composite hydrogels of each group were nontoxic to L929s and promoted the proliferation rate at low concentrations, with the highest proliferation rate occurring at a dilution of 1:8. Therefore, the 1:8 dilution extracts were chosen for subsequent experiments. RAW 264.7 cells were induced by LPS to produce intracellular ROS and then added to the composite hydrogel for coculture, and intracellular ROS were labeled using the fluorescent probe DCFH-DA to verify that BG-*x*Ce composite glass nanospheres loaded into the hydrogels also had antioxidant properties on intracellular ROS. As shown in Fig. 4A, in the control group without LPS, insufficient ROS was produced to react with the fluorescent probe and no significant green fluorescence was detected. In contrast, in the LPS group as a positive control, intracellular ROS were significantly increased and emitted intensity of green fluorescence due to LPS-induced polarization of RAW 264.7 cells toward the M1 phenotype. Compared to the LPS group, the composite hydrogel extracts reduced the number and intensity of treated cells. Figure 4B shows the quantitative statistics of the fluorescence intensity. Visually, the fluorescence intensity of all the experimental groups was less than 50% of that of the LPS group, while that of the BG-10Ce/PDA/PAM group was even less than 50% of that of other experimental groups. This result indicated the ability of the hydrogels to scavenge ROS, and the BG-10Ce/PDA/PAM group had the optimal effect.

### Regulation of macrophage polarization by composite hydrogels

**Fig. 5.**
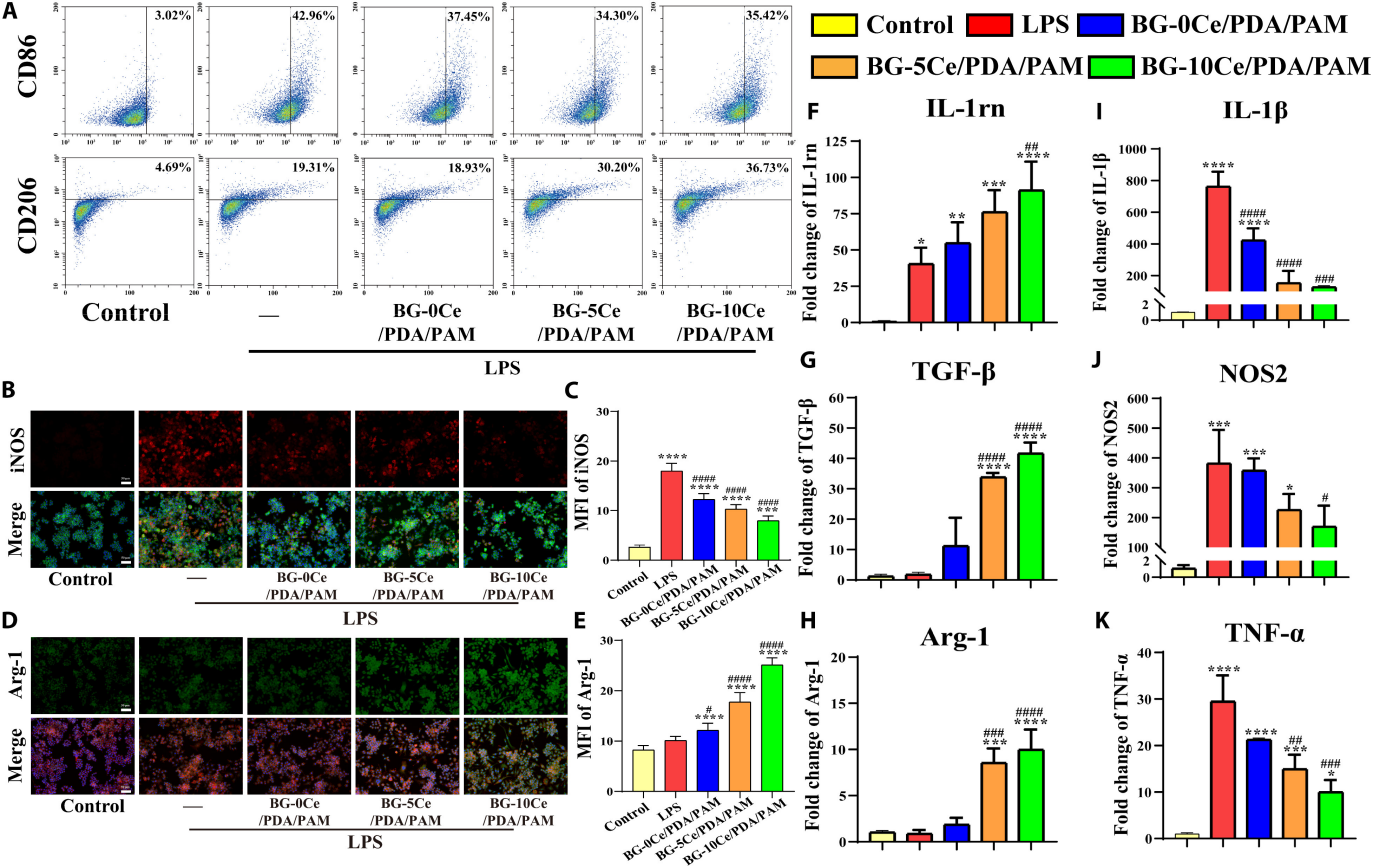
(A) Representative flow cytometry results of CD86^+^ and CD206^+^ cells. Staining images and statistics of fluorescence intensity of (B and C) M1 phenotype cytokines iNOS and (D and E) M2 phenotype cytokines Arg-1 (scale bar, 50 μm; *P versus control group, ^#^*P* versus LPS group, *P* < 0.05). Expression of proinflammatory and anti-inflammatory genes: (F) IL-1rn, (G) TGF-β, (H) Arg-1, (I) IL-1β, (J) NOS2, and (K) TNF-α (*P versus control, ^#^*P* versus LPS, *P* < 0.05).

Flow cytometry was used to measure the percentage of the surface markers CD86^+^ (M1 macrophage phenotype) and CD206^+^ (M2 macrophage phenotype). As shown in Fig. 5A, macrophages showed altered expression of M1/M2 surface markers after induction, with the positive control group showing a significantly higher percentage of CD86-positive cells than the control group. In contrast, CD206-positive cells were elevated to a certain extent in the LPS group, which might be due to negative feedback regulation caused by inflammatory cytokines secreted by M1 macrophages as a spontaneous anti-inflammatory response. The surface marker CD206^+^ for M2 macrophages was significantly higher in the BG-10Ce than in the other groups, whereas the percentage of CD86^+^ cells was significantly lower, indicating that the extract of the BG-10Ce/PDA/PAM group facilitated the polarization of macrophages toward the M2 phenotype. The quantification of flow cytometry, as shown in Fig. S9, also demonstrated this trend.

Furthermore, fluorescence staining analysis of cytokines expressed by LPS-induced macrophages after treatment with the composite hydrogel extracts was performed. High levels of proinflammation cytokines were generated by M1 macrophages, with one of the representative iNOS being selected for testing, as shown in Fig. 5B. All the extracts of the BG-*x*Ce/PDA/PAM group significantly decreased the iNOS level generated by LPS-induced macrophages, and the statistical results (Fig. 5C) also showed that the mean fluorescence intensity of the BG-10Ce/PDA/PAM group was only 48% of that of the LPS group. These findings indicated that the expression of the anti-inflammatory cytokine Arg-1 was consistent with the flow cytometry results. Specifically, the BG-10Ce/PDA/PAM group exhibited a significant increase in both the number of positive cells and the mean fluorescence intensity, promoting the polarization of M2 macrophages (Fig. 5D and E).

The results of both flow cytometry and immunofluorescence experiments supported the regulatory effect of the BG-10Ce/PDA/PAM composite hydrogels in inhibiting M1 macrophage polarization and promoting M2 macrophage polarization. The macrophage mRNA expression was evaluated to further validate this effect at the genetic level. The results indicated that the expression of proinflammatory genes related to M1 phenotype, such as IL-1rn, TGF-β, and Arg-1, was significantly decreased, while the expression of anti-inflammatory genes related to M2 phenotype, such as IL-1β, NOS2, and TNF-α, was significantly boosted (Fig. 5F to K), with the most prominent effect observed in the BG-10Ce/PDA/PAM group, alongside significant differences compared with other groups.

### Cell migration, crawling, and angiogenesis

**Fig. 6.**
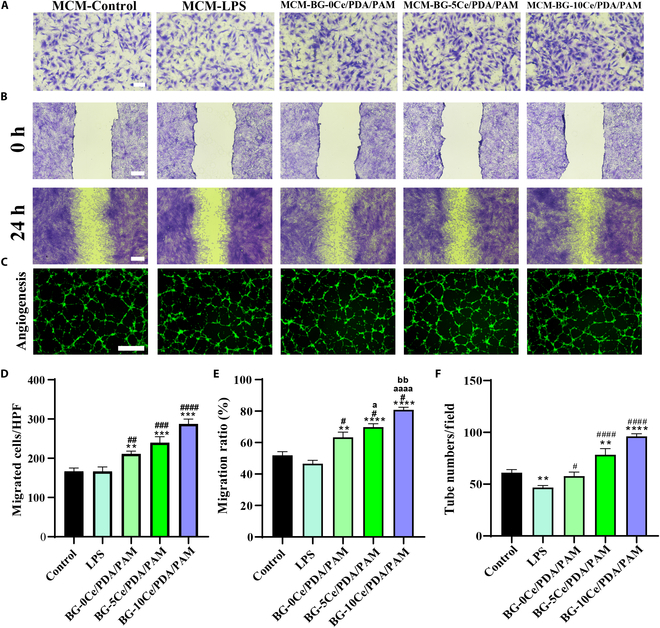
(A and D) Migration staining and migration number statistics of HUVECs in transwell plates (scale bar, 100 μm). (B and E) In vitro cell scratching assay and migration rate statistics of L929s (scale bar, 400 μm). (C and F) Image of angiogenesis assay and tube number statistics of HUVECs (scale bar, 500 μm) (^#^*P* versus control group, *P versus LPS group, ^a^*P* versus BG-0Ce group, ^b^*P* versus BG-5Ce group, *P* < 0.05).

Figure 6A and D shows the images and quantitative analysis of the migration of HUVECs to the lower chamber of the transwell under the influence of MCM. Notably, the number of cells migrating in the experiment group was higher than that in the control and LPS group. Additionally, the results of the scratch assay with L929s were comparable to those of the migration assay with HUVECs. After coculturing L929s with each group’s conditioned medium for 24 h, the scratch area was gradually filled with cells in a dense manner (Fig. 6B) in the experimental group, and fibroblast crawling was substantially enhanced. The migration rate increased as the Ce concentration in the BG-*x*Ce nanospheres increased, with the maximum value reaching 85% (Fig. 6E) and statistically significant differences compared to both the control (at 52%) and LPS groups (at 48%). The above results indicated that the BG-*x*Ce/PDA/PAM composite hydrogel promoted scratch closure, with the strongest promotion effect observed in the BG-10Ce/PDA/PAM group.

An angiogenesis assay was conducted to assess the angiogenesis of HUVECs in MCM to further elucidate the effects of BG-*x*Ce/PDA/PAM composite hydrogels on angiogenesis of endothelial cells after macrophage polarization regulation as well as ROS scavenging. As shown in Fig. 6C, the gradual formation of a closed network of endothelial cells was clearly visible in the field of view. The number of tubes also increased with increasing Ce concentration, indicating that the composite glass nanospheres promoted angiogenesis.

### Wound healing of diabetes SD rats with full-thickness skin defects

A model of full-thickness wounds in diabetes SD rats was used to evaluate the in vivo healing efficacy of the hydrogels. Before commencing the experiment, alterations in fasting blood glucose levels in SD rats after intraperitoneal injection of STZ were monitored to evaluate whether type I diabetes was effectively induced. As shown in Table S1, during the 2 weeks receiving the drug injection, all rats had blood glucose levels above 16.7 mM and were significantly different from the pre-injection values, indicating the successful modeling of rats with type I diabetes.

**Fig. 7.**
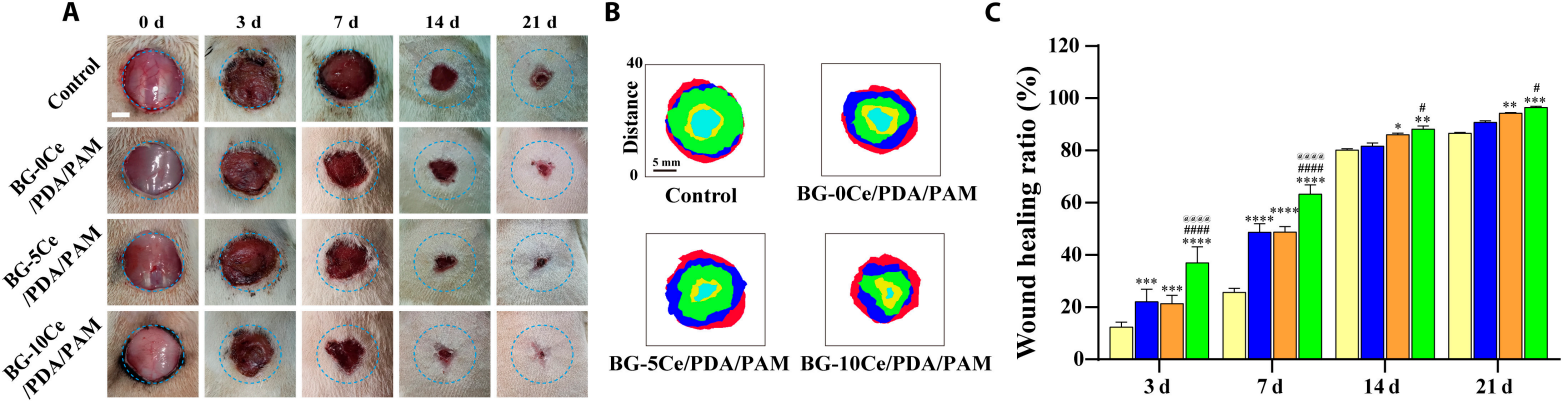
(A) Photographs of wounds in diabetes SD rats with full-thickness skin defect after treatment by control group and BG-*x*Ce/PDA/PAM composite hydrogels for 0, 3, 7, 14, and 21 d (scale bar, 5 mm). (B) Visual images of wound shrinkage process. Red represents 0 d, dark blue represents 3 d, green represents 7 d, orange represents 14 d, and light blue represents 21 d (scale bar, 5 mm). (C) Wound healing ratio for 0, 3, 7, 14, and 21 d (*P versus control group, ^#^*P* versus BG-0Ce group, ^@^*P* versus BG-5Ce group, *P* < 0.05).

As shown in Fig. 7A, the wound area was relatively uniform on day 0, after which the hydrogel in each group adhered to the wound. After 3 d of treatment, the wounds in the control group (blank hydrogel group) did not start to shrink, whereas the wounds in the experimental group (BG-*x*Ce/PDA/PAM composite hydrogel group) significantly shrank. A few granulation tissues were formed, and the wounds shrank more uniformly in the BG-10Ce/PDA/PAM group. After 7 d of treatment, the wound area in the control group showed a slight decrease, whereas that in the experimental group significantly decreased. After 21 d of treatment, the wounds in the BG-10Ce/PDA/PAM group essentially healed and recovered well, with nearly 95% than those of other groups, such as 80% of control group, 82% of BG-0Ce/PDA/PAM group, and 90% of BG-5Ce/PDA/PAM group. By plotting the area and shape of the wound at the same time point as a color gamut (Fig. 7B), the experimental groups showed significantly accelerated healing of diabetic wounds compared with the control group, with the BG-10Ce/PDA/PAM group wounds showing the fastest wound healing, indicating that the CeO_2_-NCs precipitated in the glass nanospheres could significantly accelerate wound healing. After calculating the closure rate of the diabetic wounds (Fig. 7C), the BG-10Ce/PDA/PAM group had the highest closure rate of 95% at 21 d, which was substantially different from that of the other groups.

### Histological analysis

**Fig. 8.**
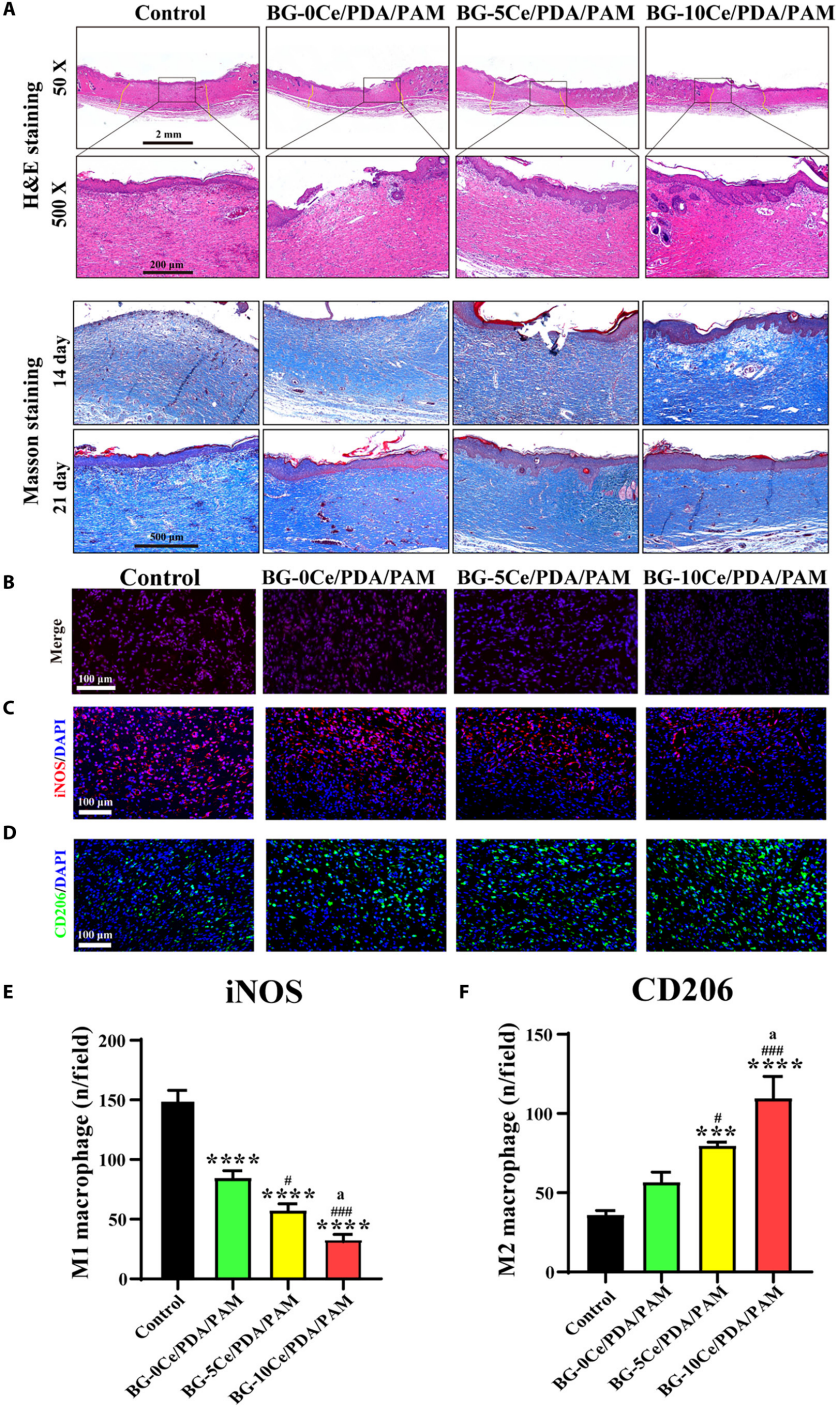
(A) Images of H&E staining [scale bar, 2 mm (50×) and 200 μm (500×)] and Masson staining (scale bar, 500 μm) for diabetic wound sections after treatment for BG-*x*Ce/PDA/PAM composite hydrogels. (B) ROS immunofluorescence staining (Merge; scale bar, 100 μm) of diabetic wound sections after treatment by BG-*x*Ce/PDA/PAM composite hydrogels. Immunofluorescence staining images of diabetic wound tissue and semiquantitative analysis of (C and E) iNOS (scale bar, 100 μm) and (D and F) CD206 (scale bar, 100 μm). (*P versus control group, ^#^*P* versus BG-0Ce/PDA/PAM group, ^a^*P* versus BG-5Ce/PDA/PAM group, *P* < 0.05).

H&E staining of slices for 21 d and Masson staining of tissue slices for 14 and 21 d were analyzed to determine the effect of the BG-*x*Ce/PDA/PAM composite hydrogel on diabetic wound healing (Fig. 8A). H&E staining revealed that the epidermis of rats in the control group was not completely closed, whereas the epidermis and dermis of rats in the experimental group were approximately normal, with a greater number of skin appendages, such as hair follicles and sebaceous tissue, than those in the control group. Masson’s trichrome staining for 14 d revealed that the control and BG-0Ce groups had less collagen deposition, whereas the blue color in sections of the BG-5Ce/PDA/PAM and BG-10Ce/PDA/PAM groups increased, indicating that collagen deposition boosted with collagen deposition was more uniform in the BG-10Ce/PDA/PAM group than in the BG-5Ce/PDA/PAM group. Collagen deposition on day 21 was denser, darker, and more uniform than on day 7.

Figure 8B and Fig. S10 show the ROS immunofluorescence staining analysis of diabetic wound sections. ROS in the wounds of the control group maintained a very high fluorescence density and intensity, but the fluorescence intensity of the experimental group decreased as the Ce concentration of the BG-*x*Ce glass nanospheres increased, while the BG-10Ce/PDA/PAM group exhibited the lowest local residual ROS fluorescence staining intensity. These results indicated that the BG-10Ce/PDA/PAM group had the strongest ability to scavenge oxidative stress, which was consistent with the results of the in vitro antioxidant tests. Moreover, this proved that the CeO_2_-NCs precipitated on the glass nanospheres could enhance the antioxidant effect.

iNOS was the M1 macrophage marker, and CD206 was the M2 macrophage marker. Local tissue sections were analyzed using immunofluorescence staining for iNOS and CD206. In the experimental group, the fluorescence area and fluorescence intensity of iNOS were significantly reduced (Fig. 8C and E), and the fluorescence intensity of CD206 (Fig. 8D and F) was significantly increased, indicating that BG-10Ce/PDA/PAM could significantly regulate local macrophage polarization to the M2 phenotype and inhibit M1 polarization.

**Fig. 9.**
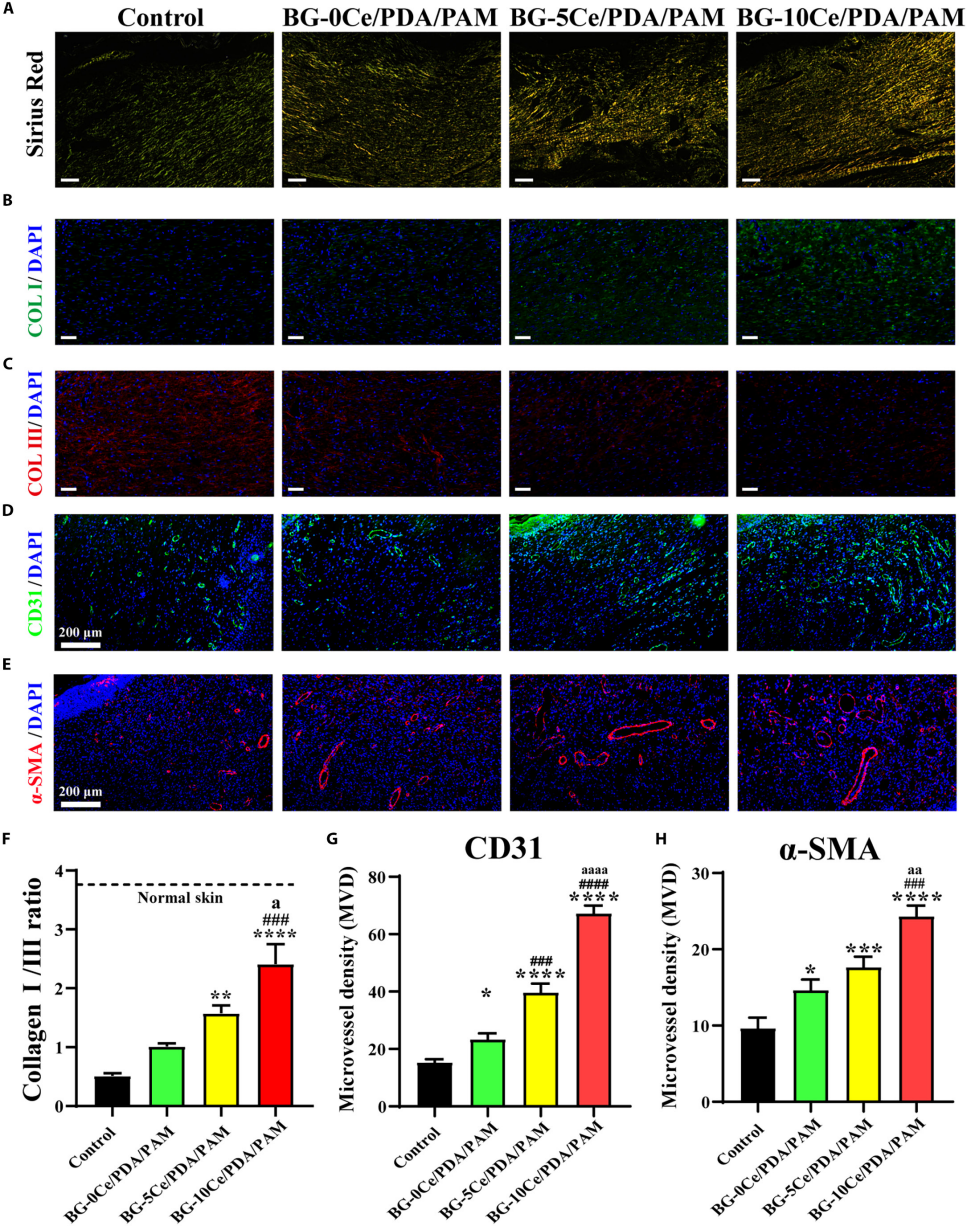
(A) Sirius red staining of diabetic wound tissue on day 21. Orange demonstrates collagen I, and green demonstrates collagen III (scale bar, 200 μm). Immunofluorescence staining images of diabetic wound tissue of (B) collagen I (scale bar, 50 μm), (C) collagen III (scale bar, 50 μm), (D) CD31 (scale bar, 200 μm), and (E) α-SMA (scale bar, 200 μm). Semiquantitative analysis of (F) collagen I/III ratio, (G) CD31, and (H) α-SMA. (*P versus control group, ^#^*P* versus BG-0Ce/PDA/PAM group, ^a^*P* versus BG-5Ce/PDA/PAM group, *P* < 0.05).

Sirius red staining and immunofluorescence staining were also performed to study the arrangement and composition of the collagen in the regenerated skin tissue. Type III collagen is produced in the early phase and gradually replaced by type I collagen, which is responsible for tissue structural stability [43]. Alterations in the ratio of collagen type I/III ratio are hallmark indicators of injury and aging in the skin, which makes this result particularly significant [44]. As shown in Fig. 9A to C, on day 21, the BG-10Ce/PDA/PAM group contained large numbers of regularly arranged collagen fibers and had a significantly higher collagen I/III ratio (at 2.41) than those of other group, such as BG-0Ce/PDA/PAM group of 1.01 and BG-5Ce/PDA/PAM group of 1.58 (Fig. 9F). The results demonstrated that the BG-10Ce/PDA/PAM group could accelerate the process of transforming immature granulation tissue into mature skin tissue and had the best therapeutic effect on the diabetic wound. Furthermore, immunofluorescence staining of CD31 and α-SMA was analyzed to evaluate the blood vessel regeneration. As shown in Fig. 9D and E, the fluorescence intensity of CD31 and α-SMA increased as the Ce concentration of BG-*x*Ce glass nanospheres increased, indicating that the amount of neovascularization in the wounds area progressively increased. The above results might be due to 2 reasons: Ions released from BG-*x*Ce composite glass nanospheres, such as B, could significantly accelerate wound closure and promote angiogenesis. In contrast, CeO_2_-NCs scavenged local oxidative stress and restored the vascular regeneration of local tissues, thus promoting an increase in local blood vessels.

## Discussion

Compared to normal patients, diabetes patients exhibit an unbalanced inflammatory response, excessive oxidative stress, and lower angiogenic capacity, resulting in chronic and hard-to-heal wounds [45]. Therefore, the development of multifunctional diabetic wound dressings with antioxidant properties has become one of the most attractive strategies for accelerating the restoration of chronic wounds. CeO_2_-NCs have been demonstrated to have tremendous potential for diabetic wound healing applications [20,46,47], owing to the high biocompatibility and low toxicity and leading to minimal local tissue reactions [48]. As nanozymes, CeO_2_-NCs can remove excessive ROS, which is related to their nonstoichiometric composition accompanied by oxygen vacancies [49,50]. Moreover, the CeO_2_-NCs involved in the redox process can be restored to their initial state within a relatively short period [51], which is more effective than Ce ions in scavenging ROS. However, free CeO_2_-NCs are easily excreted by organisms [52], and phagocytosis of CeO_2_-NCs by cells can result in significant cytotoxic effects [53], limiting their application in biomedicine as antioxidants. Therefore, combining CeO_2_-NCs with other materials can maximize their benefits for diabetes-related regeneration [54].

Sr/Ce-doped mesoporous borosilicate BG was prepared using the sol–gel method. After carrying out a proper heat treatment, BG-*x*Ce composite glass nanospheres were obtained, in which CeO_2_-NCs were effectively deposited on the surface. According to the XRD results, CeO_2_-NCs with specific structures were not formed in the BG-5Ce composite glass nanospheres, in which Ce was embedded in the network structure of the glass. CeO_2_-NCs precipitated on the surfaces of the nanospheres when the Ce content reached 10 mol%. However, the precipitation was not dense; rather, it covered the surface of the nanospheres, further increasing the SSA and pore volume (Fig. 2B and C). Therefore, BG-10Ce had the highest antioxidant effect than other groups (Figs. S4 and S5). CeO_2_-NCs attached to the surface of the nanospheres could continuously scavenge ROS from the wound without being metabolized or endocytosed by the cells.

A good adhesive performance can prevent dressing from falling off wounds and ensures long-term performance [25]. Compared with that of the hydrogel without glass nanospheres, the adhesive results of the BG-*x*Ce/PDA/PAM composite hydrogel demonstrated a decrease in the adhesive strength of the composite hydrogel, which was ascribed to the o-phenyl hydroxyl group in PDA being an important source of hydrogel adhesion. In general, DA reacts with oxygen in air and APS in an alkaline environment via oxidation, gradual cross-linking, and consumption of the o-phenyl hydroxyl group to generate o-phenyl quinone, leading to a decrease in hydrogel adhesion. The degradation of glass nanospheres in solution caused an alkaline environment (pH = 8.6 ± 0.2 in the BG-0Ce/PDA/PAM group, pH = 8.7 ± 0.1 in the BG-5Ce/PDA/PAM group, and pH = 8.3 ± 0.2 in the BG-10Ce/PDA/PAM group), which accelerated the DA oxidation reaction and reduced the adhesive strength of the BG-0Ce/PDA/PAM group. However, CeO_2_-NCs could act as a reductant and limit the degree of the oxidation reaction; therefore, the composite hydrogel retained more o-phenylene hydroxyl groups, which retained the adhesion of the BG-5Ce/PDA/PAM (56.3 kPa) and BG-10Ce/PDA/PAM (60.84 kPa) groups to a certain extent compared with the BG-0Ce/PDA/PAM group (46.5 kPa).

When skin is damaged, fibroblasts play a crucial role in wound closure by accumulating and migrating from the area adjacent to the wound. Simultaneously, normal wound healing depends on the neovascularization and continuous growth into the center of the wound, which is closely related to the migration and angiogenic activity of endothelial cells. However, excessive ROS accumulates at the wound site due to the detrimental microenvironment of diabetic wounds, which has a damaging impact on cells or tissues and results in excessive oxidative stress. Meanwhile, excessive oxidative stress will impair the function of dermal fibroblasts and keratinocytes, leading to excessive persistent inflammation and inadequate angiogenic capacity, which can have serious consequences that make it difficult for the wound to heal [55]. Therefore, wound dressings must be able to scavenge ROS in diabetic wounds and reduce local inflammatory responses to ensure that the wound microenvironment is normalized, promoting angiogenesis and tissue regeneration. We explored the ability of the BG-*x*Ce/PDA/PAM composite hydrogels to scavenge intracellular ROS and modulate macrophage polarization. Fluorescence staining of in vitro cells and diabetic wound tissue section revealed that all 3 groups of composite hydrogels could scavenge ROS, with the BG-10Ce/PDA/PAM group exhibiting the strongest scavenging ability (Figs. 4 and 8B). This was due to the SOD and CAT-like activities of the CeO_2_-NCs, which catalyzed the decomposition of intracellular superoxide anions to further reduce intracellular ROS [51]. Moreover, the BG-0Ce/PDA/PAM group also had the ability to scavenge intracellular ROS, probably because Sr ions dissolved during the degradation of the composite glass nanospheres modulated macrophage polarization and reduced the production of ROS in macrophages. The healing of diabetic wounds stagnates in the inflammation stage, which is mediated by macrophages. The phenotypic transformation of macrophages from M1 (proinflammatory phenotype) to M2 (anti-inflammatory phenotype) is essential for promoting wound healing from the inflammation stage to the proliferation stage [25]. M2 macrophages secrete a variety of cytokines and growth factors [epidermal growth factor (EGF), fibroblast growth factor (FGF), and vascular endothelial growth factor (VEGF)] that promote cell proliferation, differentiation, and ECM deposition, promoting wound healing process of wounds, contributing to the maturation of regenerative tissue, and reorganizing the ECM to eliminate scar tissue [56]. The results of in vitro experiments on macrophage modulation by composite hydrogels revealed that the expression levels of the M2 macrophage marker CD206 (Fig. 5A) and the corresponding anti-inflammatory genes (Fig. 5I to K) were significantly higher in the BG-10Ce/PDA/PAM group than in the other groups that the BG-10Ce/PDA/PAM group significantly promoted the polarization of macrophages toward the M2 phenotype, which has also been confirmed in animal studies (Fig. 8C to F). This is because Sr is an important element that regulates the polarization phenotype of macrophages [23,57], and the BG-10Ce composite glass nanospheres led to a higher release of Sr than the other groups because of the larger amount of introduced Ce (Fig. S2C). Thus, the corresponding hydrogels could effectively regulate the polarization of macrophages toward the M2 phenotype. Furthermore, full-thickness skin defect experiment and collagen I/III ratio also proved that diabetic wound healing was accelerated at the normal microenvironment (Figs. 7 and 9).

The BG-*x*Ce/PDA/PAM composite hydrogel demonstrated a dual function in diabetic wound healing, with the CeO_2_-NCs attached to the composite glass nanospheres acting as nanozymes that catalyze the decomposition of ROS in cells and wounds, reduce oxidative stress, and establish a wound microenvironment that is conducive to cell migration and angiogenesis. Meanwhile, during the degradation process, the glass nanospheres released functional ions, such as Sr and B, which could regulate the polarization of macrophages to the M2 phenotype, and reduce the inflammatory response at the wound site, while significantly promoting angiogenesis and wound healing. The synergy between the amorphous phase and nanocrystals can effectively promote the healing of diabetic wounds.

### Conclusion

In this study, a BG-*x*Ce/PDA/PAM composite hydrogel with good adhesion and tensile properties was prepared; CeO_2_-NCs participated on the surface of BG-10Ce composite glass nanospheres after proper heat treatment and could significantly scavenge intracellular ROS and reduce oxidative stress. Meanwhile, ions released from the BG-*x*Ce composite glass nanospheres regulated the polarization of macrophages toward the M2 phenotype, promoting cell migration and angiogenesis. The synergistic effect of BG and CeO_2_-NCs can promote the healing of diabetic wounds and provide a new option for diabetic wounds.

## Ethical Approval

The study was approved by the Ethics Committee of the Institutional Animal Care and Use Committee of Shanghai Jiao Tong University Affiliated Sixth People’s Hospital (permit number DWSY2022-0028).

## Data Availability

Data will be made available on request.
