# Supplementary material for: Diphasic CeO2 Nanocrystal/Bioactive Glass Nanosphere-Based Composite Hydrogel for Diabetic Wound Healing by Reactive Oxygen Species Scavenging and Inflammation Regulation
Source: Biomater Res. 2024 Sep 16;28:0066. doi: 10.34133/bmr.0066 (PMC11403469; doi:10.34133/bmr.0066)
Supplement: Supplementary 1 — Figs. S1 to S10 Table S1 [file bmr.0066.f1.docx]

**Diphasic CeO_2_ nanocrystal/bioactive glass nanosphere based composite hydrogel for diabetic wound healing by ROS scavenging and inflammation regulation**

Muyan Qin ^a, 1^, Ziyang Zhu ^b, 1^, Jingxin Ding ^a, 1^, Jinhui Zhao ^b, c, d^, Lingtian Wang ^b^, Dajun Jiang ^b^, Deping Wang ^a, *^, Weitao Jia ^b, *^

^a^ School of Materials Science and Engineering, Tongji University, Shanghai, 201804, China.

^b^ Department of Orthopedic, Shanghai Jiao Tong University Affiliated Sixth People's Hospital, Shanghai 200233, China.

^c^ Department of Orthopedics, Shanghai Tenth People's Hospital, School of Medicine, Tongji University, Shanghai, China.

* Corresponding authors’ email addresses: [wdpshk@tongji.edu.cn](mailto:wdpshk@tongji.edu.cn), jiaweitao@shsmu.edu.cn

^1^ These authors contributed equally to this work.

**1. Supporting Figures**


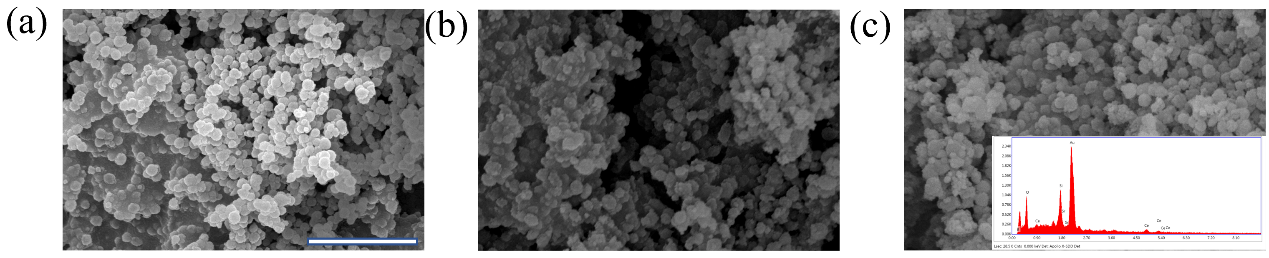


**Fig. S1**. SEM images of BG-xCe (x = 0, 5, 10 mol%) composite glass nanospheres, where (c) the EDS energy spectrum of the built-in figure BG-10Ce


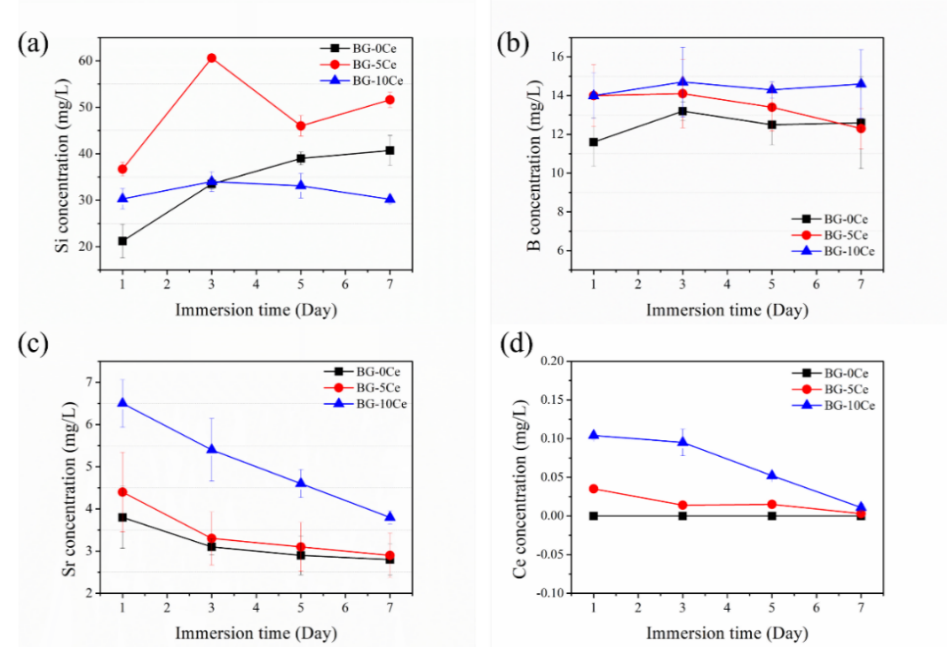


**Fig. S2**. Ion release concentration profiles of BG-xCe composite glass nanospheres immersed in PBS solution for 1, 3, 5 and 7 days: (a) Si, (b) B, (c) Sr, and (d) Ce


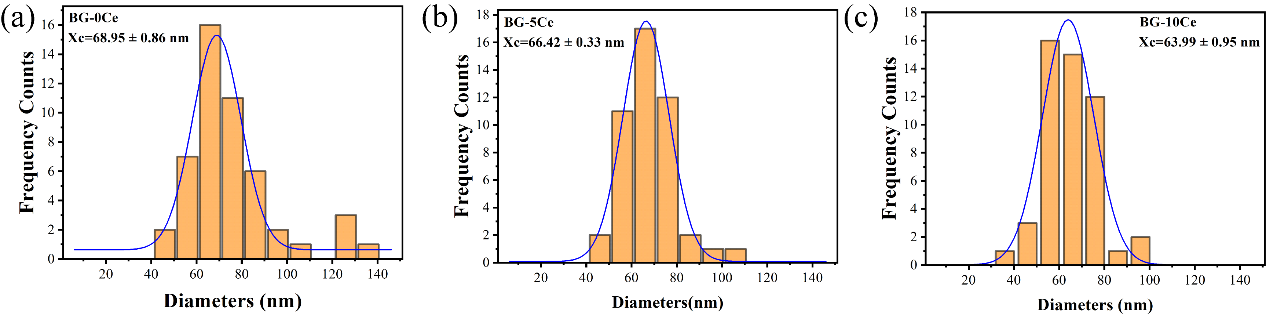


**Fig. S3**. Particle size distribution statistics of BG-xCe (x=0, 5, and 10 mol%) composite glass nanospheres


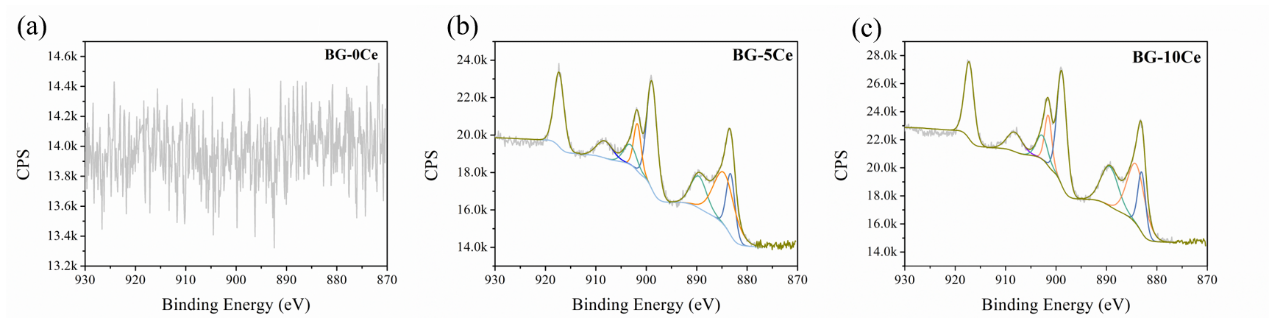


**Fig. S4**. XPS pattern analysis of Ce in BG-xCe composite glass nanospheres: (a) BG-0Ce, (b) BG-5Ce, (c) BG-10Ce. Brown lines can be attributed to Ce^3+^ and blue lines to Ce^4+^.


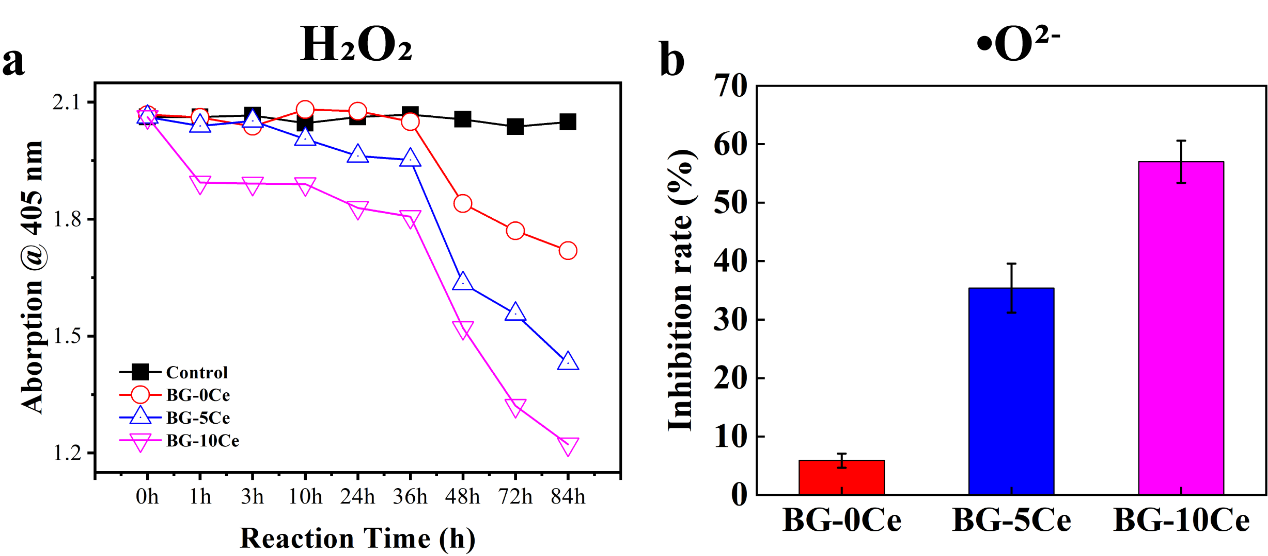


**Fig. S5**. (a) The quantitative estimation of H_2_O_2_ scavenging capacity of glass nanospheres (n = 5). (b) The quantitative evaluation of •O^2-^ scavenging capacity of glass nanospheres. (n = 5).


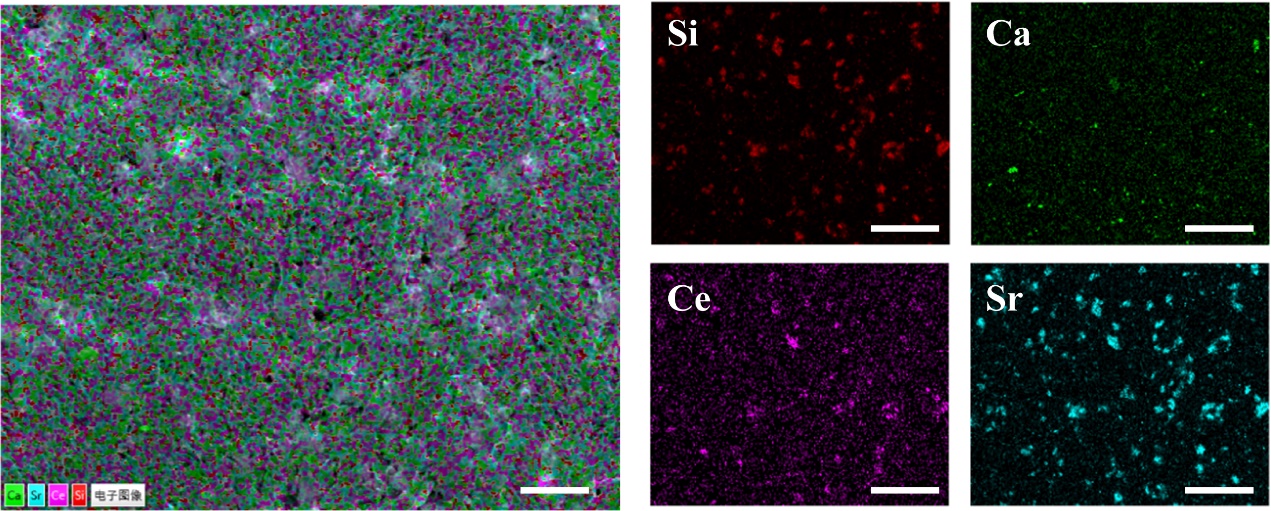


**Fig. S6**. Elemental images (Si, Ca, Ce, Sr) of BG-10Ce/PDA/PAM composite hydrogels (Scale bar = 500 μm)


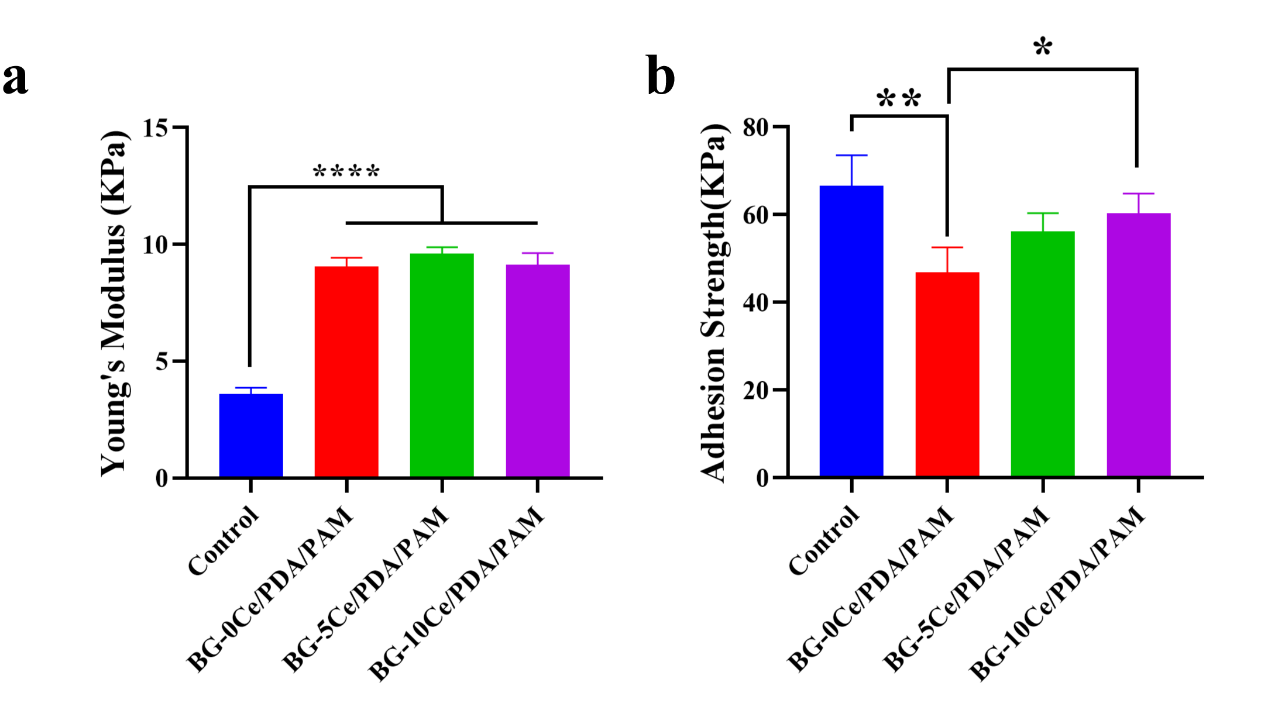


**Fig. S7**. (a)Young’s modulus and (b) adhesion strength of BG-xCe/PDA/PAM composite hydrogel. (*P < 0.05)


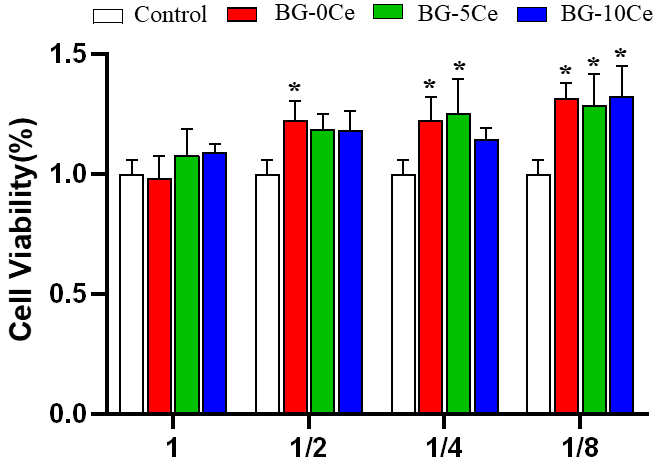


**Fig. S8**. The proliferation of L929s after treatment by hydrogels. (*P vs Control, P < 0.05)

**
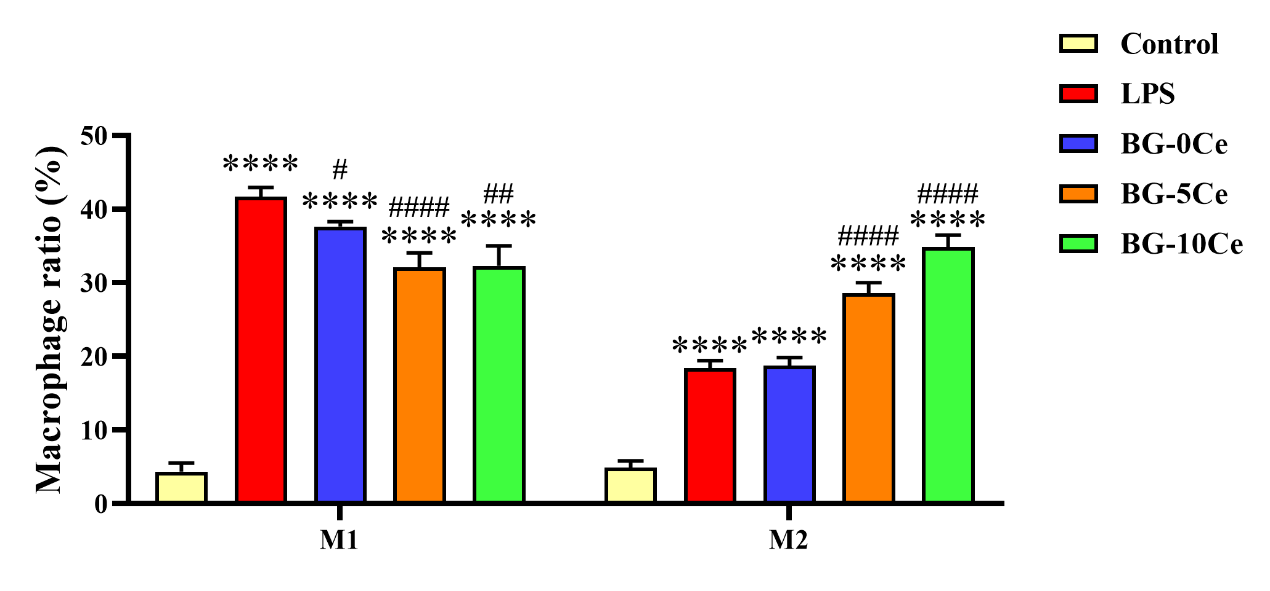
****Fig. S9**. The quantification of flow cytometry. (*P vs Control group, **^#^**P vs LPS group, P < 0.05)


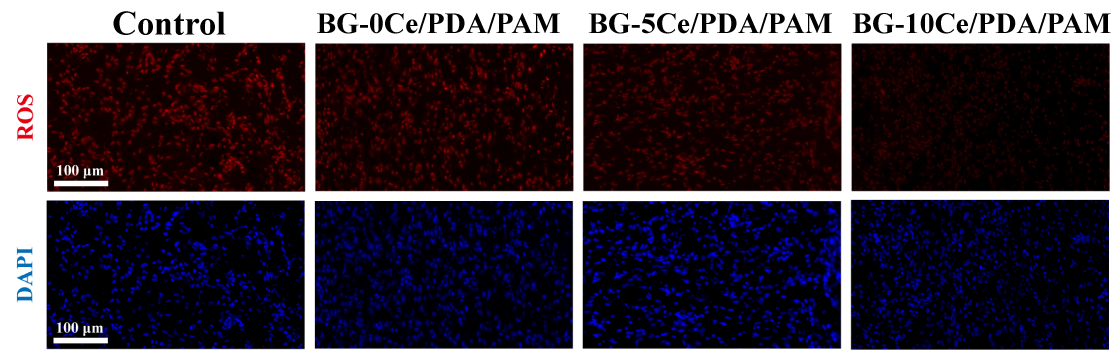


**Fig. S10.** ROS immunofluorescence staining (ROS and DAPI, Scale bar = 100 μm) of diabetic wounds sections after treatment by BG-xCe/PDA/PAM composite hydrogels.

**2. Supporting Tables**

**Table S1**. Mean blood glucose levels in all diabetic rats before the experiment

| Time | 0 d | 3 d | 7 d | 10 d | 14 d |
| --- | --- | --- | --- | --- | --- |
| Mean blood glucose level (mmol/L) | 6.63 | 22.48 | 21.58 | 23.31 | 26.59 |
| Error (mmol/L) | 0.81 | 2.54 | 1.35 | 2.17 | 4.36 |
